# Molecular Characterization of Nine TRAF Genes in Yellow Catfish (*Pelteobagrus fulvidraco*) and Their Expression Profiling in Response to *Edwardsiella ictaluri* Infection

**DOI:** 10.3390/ijms24098363

**Published:** 2023-05-06

**Authors:** Shen-Li You, Xin-Xin Jiang, Gui-Rong Zhang, Wei Ji, Xu-Fa Ma, Xu Zhou, Kai-Jian Wei

**Affiliations:** Key Laboratory of Freshwater Animal Breeding, Ministry of Agriculture and Rural Affairs, College of Fisheries, Huazhong Agricultural University, Wuhan 430070, China

**Keywords:** TRAFs, molecular characterization, *Edwardsiella ictaluri*, mRNA expression, subcellular localization

## Abstract

The yellow catfish (*Pelteobagrus fulvidraco*) is an economic fish with a large breeding scale, and diseases have led to huge economic losses. Tumor necrosis factor receptor-associated factors (TRAFs) are a class of intracellular signal transduction proteins that play an important role in innate and adaptive immune responses by mediating NF-κB, JNK and MAPK signaling pathways. However, there are few studies on the TRAF gene family in yellow catfish. In this study, the open reading frame (ORF) sequences of TRAF1, TRAF2a, TRAF2b, TRAF3, TRAF4a, TRAF4b, TRAF5, TRAF6 and TRAF7 genes were cloned and identified in yellow catfish. The ORF sequences of the nine TRAF genes of yellow catfish (*Pf_*TRAF1-7) were 1413–2025 bp in length and encoded 470–674 amino acids. The predicted protein structures of *Pf_*TRAFs have typically conserved domains compared to mammals. The phylogenetic relationships showed that TRAF genes are conserved during evolution. Gene structure, motifs and syntenic analyses of TRAF genes showed that the exon–intron structure and conserved motifs of TRAF genes are diverse among seven vertebrate species, and the TRAF gene family is relatively conserved evolutionarily. Among them, TRAF1 is more closely related to TRAF2a and TRAF2b, and they may have evolved from a common ancestor. TRAF7 is quite different and distantly related to other TRAFs. Real-time quantitative PCR (qRT-PCR) results showed that all nine *Pf_*TRAF genes were constitutively expressed in 12 tissues of healthy yellow catfish, with higher mRNA expression levels in the gonad, spleen, brain and gill. After infection with *Edwardsiella ictaluri*, the expression levels of nine *Pf_*TRAF mRNAs were significantly changed in the head kidney, spleen, gill and brain tissues of yellow catfish, of which four genes were down-regulated and one gene was up-regulated in the head kidney; four genes were up-regulated and four genes were down-regulated in the spleen; two genes were down-regulated, one gene was up-regulated, and one gene was up-regulated and then down-regulated in the gill; one gene was up-regulated, one gene was down-regulated, and four genes were down-regulated and then up-regulated in the brain. These results indicate that *Pf_*TRAF genes might be involved in the immune response against bacterial infection. Subcellular localization results showed that all nine *Pf_*TRAFs were found localized in the cytoplasm, and *Pf_*TRAF2a, *Pf_*TRAF3 and *Pf_*TRAF4a could also be localized in the nucleus, uncovering that the subcellular localization of TRAF protein may be closely related to its structure and function in cellular mechanism. The results of this study suggest that the *Pf_*TRAF gene family plays important roles in the immune response against pathogen invasion and will provide basic information to further understand the roles of TRAF gene against bacterial infection in yellow catfish.

## 1. Introduction

Tumor necrosis factor receptor-associated factors (TRAFs) are a class of genetically conserved and critical intracellular adaptor proteins that were first discovered in mammals [1,2]. They were initially considered as a class of signal adapters that activate downstream signals by binding directly to the cytoplasmic domain of tumor necrosis factor receptors (TNFR) [3,4,5]. TRAF1 and TRAF2 were first identified and named in 1994, and since then TRAFs have been studied intensively [6]. A total of seven TRAFs have been identified in mammals, namely TRAF1–7 [7,8]. Except for TRAF7, TRAFs have similar structural features, including the TRAF domain, a RING finger and a zinc finger [6,9,10]. TRAF1–6 contain a C-terminal TRAF domain with high similarity, which is considered the basis for recognizing the TRAF family [9,11,12]. The TRAF domain can be divided into two subdomains (the MATH domain and the coiled-coil structure region), which mediate TRAF oligomerization and interaction with upstream regulators and downstream effectors [9,11]. The C-terminal domain of the TRAF7 is replaced by seven WD40, which act similarly to the TRAF domains [8,13]. The N-terminal domain of TRAFs is composed of RING finger and zinc finger domains, except TRAF1. The RING finger domain is found in many E3 ubiquitin ligases and is important for regulating downstream pathways and mediating the activation of NF-κB [1,14]. The zinc finger domain is involved in activating NF-κB and JNK pathways by mediating DNA binding and protein interactions [15].

TRAFs play an important role in regulating innate and adaptive immunity, embryonic development, stress response, tissue homeostasis and bone metabolism [16,17]. The TRAFs can directly or indirectly bind to the death domain (TRADD or FADD) or the TRAF binding domain of the TNFR [12]. TRAF-mediated innate immunity mainly includes Toll-like receptor (TLR), NOD-like receptor (NLR) and RIG-I-like receptor (RLR) pathways [18,19,20,21,22,23]. TRAF3 and TRAF6 can participate in the TLR signaling pathway by interacting with the downstream proteins of the TLR (MyD88 and TRIF) during pathogens invasion, eventually inducing cells to produce chemokines, type I interferons, pro-inflammatory cytokines and antimicrobial enzymes [18,19], while TRAF4 can block the binding of TRAF6 to TRIF and disrupt TLR signaling [20]. TRAF1, TRAF2, TRAF5 and TRAF6 can transmit signals by binding to the downstream protein RIP2 of NOD1 and NOD2, which are important members of the NLR family [21]. NLRP3 of the NLR family can induce the secretion of inflammasome under the regulation of TRAF3 [22]. TRAF4 can directly bind to NOD2 and is phosphorylated by IKKa, which inhibits the activity of NF-κB and negatively regulates NLR [23]. The RLR family is a family of intracellular RNA recognition receptors and has three members, RIG-1, MDA-5 and LGP2 [15]. Upon detection of viral RNA in the cytosol, RLR dimerizes and binds to mitochondrial antiviral signaling protein (MAVS) [24,25]. TRAFs can combine with activated MAVS to activate NF-κB signaling via the coiled-coil domains [26,27,28].

As in mammals, teleosts also possess TRAFs. The study on teleost TRAFs mainly focuses on the isolation and identification of gene sequences. Most teleosts lack the TRAF1 and TRAF5 genes. TRAF2–7 genes have been found in Chinese tongue sole (*Cynoglossus semilaevis*) [29] and black rockfish (*Sebastes schlegelii*) [30], among which the TRAF2 gene in Chinese tongue sole has TRAF2a and TRAF2b isoforms, and black rockfish has an additional TRAF2a-like gene. TRAFs’ proteins of both Chinese tongue sole and black rockfish have RING finger domains, TRAF2–6 contain one MATH domain and different numbers of zinc finger domains, and TRAF7 has seven consecutive WD40 domains [29,30]. Notably, TRAF5 of black rockfish does not have a MATH domain [30]. The gene structures of TRAF3, TRAF4 and TRAF6 in Nile tilapia (*Oreochromis niloticus*) also conformed to the above characteristics [31,32,33]. The mRNA expression analyses of TRAF genes in the healthy tissues of the three fish species indicate that TRAFs have high mRNA levels in the gill; TRAF2a, TRAF4 and TRAF6 mRNAs are highly expressed in the brain; and TRAF3 and TRAF7 mRNAs are highly expressed in the spleen. TRAF4 is closely related to embryonic development [29,30,31,32,33]. In Nile tilapia, after *Streptococcus agalactiae* infection, the mRNA expression of TRAF3 was significantly up-regulated in the head kidney, gill, spleen and skin [33]. The mRNA expression of TRAF4 in the intestine of Nile tilapia was significantly down-regulated, and the expression levels of TRAF4 mRNA in the gill and blood and TRAF6 mRNA in the intestine were first down-regulated and then up-regulated [31,32]. The mRNA levels of TRAFs (except TRAF7) were significantly up-regulated in the gill and spleen of Chinese tongue sole after infection with *Vibrio harvei* [29] and in the liver of black rockfish after infection with *Vibrio anguillarum* [30]. The changes in TRAFs mRNA levels in gill were quite variable after bacterial infection. The mRNA levels of TRAF3, TRAF5 and TRAF7 in black rockfish and those of TRAF7 in Chinese tongue sole were down-regulated after bacterial infection, while there was an up-regulation phenomenon in the mRNA level of other TRAFs [29,30]. In addition, there have been a few studies on the TRAF genes in zebrafish [34] and grass carp [35]. These results indicate that the TRAFs participate in the immune response of gill and other immune-related tissues in teleosts and play a role in resisting pathogen infection. However, there is limited information on the role of TRAFs in yellow catfish.

Yellow catfish (*Pelteobagrus fulvidraco*) is an important commercial fish in China. With the increase in culture area and density, bacterial and parasitic diseases frequently outbroke and caused serious economic losses [36]. Compared to the specific immune system, the innate immune system is less limited and plays an important role in the anti-bacterial infection process [37]. As an important intracellular signal transduction protein, TRAF plays an important role in the process of anti-bacterial infection [24]. Although the TRAF gene family has been found in a variety of fish, there is still a lack of systematic research on the TRAF gene family in yellow catfish. It is essential to explore the role of *Pf_*TRAF genes in the immune response against bacterial infections for the prevention and treatment approaches of bacterial diseases in yellow catfish. This study aims to (1) clone the open reading frame (ORF) sequences of *Pf_*TRAF genes from yellow catfish and probe into the protein structure characterizations and evolutionary relationships of *Pf_*TRAFs; (2) detect the mRNA expressions of *Pf_*TRAF mRNAs in various tissues of healthy yellow catfish and the expression changes of *Pf_*TRAF mRNAs in several immune-related tissues after infection by *Edwardsiella ictaluri*; and (3) investigate the subcellular localization of *Pf_*TRAFs in HEK293T cells was investigated by constructing the TRAFs-pEGFP-N1 eukaryotic vector. The results of this study will provide basic information to better understand the role of *Pf_*TRAF genes in the immune response against bacterial infection in yellow catfish and provide theoretical reference for the prevention and treatment of bacterial diseases in the process of the cultivation of yellow catfish.

## 2. Results

### 2.1. Protein Structural Characterization of Pf_TRAF1–7

A total of nine TRAF genes were cloned and identified from yellow catfish, including *Pf_*TRAF1, *Pf_*TRAF2a, *Pf_*TRAF2b, *Pf_*TRAF3, *Pf_*TRAF4a, *Pf_*TRAF4b, *Pf_*TRAF5, *Pf_*TRAF6 and *Pf_*TRAF7. The nine TRAF genes had ORF lengths of 1413 bp (*Pf_*TRAF4a) to 2025 bp (*Pf_*TRAF7) and encoded 470 amino acids (aa) (*Pf_*TRAF4a) to 674 aa (*Pf_*TRAF7) (Table 1). The predicted MWs of the nine *Pf_*TRAF proteins were between 54.16 kDa (*Pf_*TRAF4a) and 74.86 kDa (*Pf_*TRAF7), with estimated pI values ranging from 6.45 (*Pf_*TRAF6) to 8.26 (*Pf_*TRAF4b) (Table 1). The predicted *Pf_*TRAF2b and *Pf_*TRAF4b proteins contained two N-glycosylation sites (*Pf_*TRAF2b: NFSV^84–87^ and NLSN^332–335^; *Pf_*TRAF4b: NGTS^329–332^ and NGSG^357–360^), the predicted *Pf_*TRAF1, *Pf_*TRAF2a, *Pf_*TRAF4a, *Pf_*TRAF5 and *Pf_*TRAF6 proteins contained one N-glycosylation site (*Pf_*TRAF1: NVSQ^401–404^; *Pf_*TRAF2a: NXTV^90–93^; *Pf_*TRAF4a: NGNG^353–356^; *Pf_*TRAF5: NRTL^32–35^; and *Pf_*TRAF6: NVSC^131–134^) and the predicted *Pf_*TRAF3 and *Pf_*TRAF7 proteins have no N-glycosylation site (Supplementary Figure S1).

**Figure 1:**
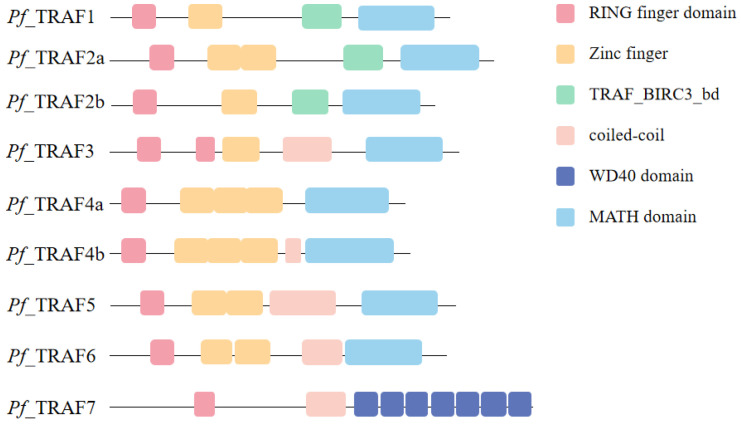
Conserved domains of the *Pf_*TRAF proteins. Protein domains are shown relative to the length of the position within the amino acid sequences.

The predicted *Pf_*TRAF3 protein contained two RING finger structures, while other *Pf_*TRAF proteins had one RING finger structure in their N-terminal region (Figure 1). Meanwhile, the *Pf_*TRAF1–6 proteins all contained a MATH domain, while the *Pf_*TRAF7 protein had seven continuous WD40 structures in its C-terminal region. The *Pf_*TRAF1–6 proteins all had one to three canonical zinc finger domains. In addition, the *Pf_*TRAF1, *Pf_*TRAF2a and *Pf_*TRAF2b proteins possessed the TRAF_BIRC3_bd domain, while the *Pf_*TRAF3, *Pf_*TRAF4b, *Pf_*TRAF5, *Pf_*TRAF6, and *Pf_*TRAF7 proteins had a coiled-coil structure (Figure 1).

### 2.2. Gene Structure and Motif Composition Analysis

To further understand the structural diversity of the TRAF family genes, the exons and introns of vertebrate TRAF genes were analyzed (Supplementary Figure S2) by consulting the NCBI database. Teleost TRAF1, vertebrate TRAF2, vertebrate TRAF3 and vertebrate TRAF5 all had 10 exons and 9 introns, except chicken TRAF2 and rainbow trout TRAF5 which had 11 exons and 10 introns. Vertebrate TRAF4 had seven exons and six introns, except zebrafish TRAF4 which had eight exons and seven introns. Vertebrate TRAF6 had six exons and five introns, except rainbow trout TRAF6 which had seven exons and six introns. In contrast, teleost TRAF7 had 18–22 exons, and tetrapod TRAF7 had 20 exons and 19 introns.

**Figure 2:**
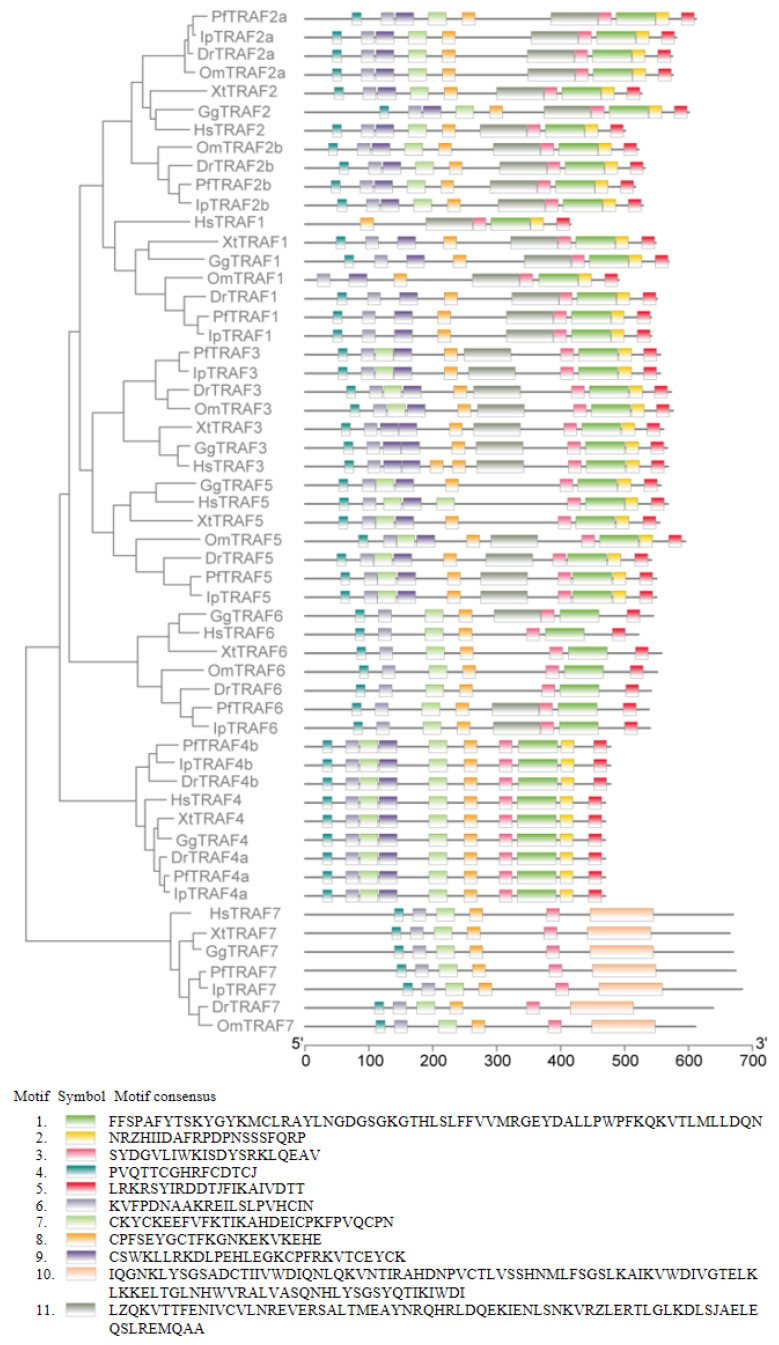
Analysis of conserved motifs in TRAF proteins. Conserved motif analysis of TRAF proteins from seven different vertebrate species: *Homo sapiens*, *Gallus gallus*, *Xenopus tropicalis*, *Oncorhynchus mykiss*, *Danio rerio*, *Ictalurus punctatus* and *Pelteobagrus fulvidraco*. The conserved motifs were identified with MEME software. The settings were as follows: maximum number of different motifs, 11; minimum motif width, 6; and maximum motif width, 100. The lengths of the motifs are shown proportionally. Eleven putative motifs are indicated by different colored boxes. The GenBank accession numbers of the TRAF amino acids used in the motif composition are listed in Supplementary Table S2.

The MEME Suite web server was used to identify conserved motifs of TRAF proteins from yellow catfish and the above species, and 11 conserved motifs were found with motif lengths ranging from 15 to 100 (Figure 2). Motif 3 was found in all TRAF1–7 proteins of seven vertebrates, and motifs 4, 6 and 8 were present in almost all TRAF1–7 proteins (except for the human TRAF1 protein lacking motifs 4 and 6, the rainbow trout TRAF1 protein lacking motif 4, and the human TRAF5 protein lacking motif 8). Motifs 1 and 5 were found in all TRAF1–6 proteins, motifs 2 and 9 were found in almost all TRAF1–5 proteins (except for the human TRAF1 protein lacking motif 9), motif 7 was found in almost all TRAF2–7 proteins (except for the tetrapod TRAF3 protein lacking motif 7), and motif 10 was found only in TRAF7 protein. Additionally, motif 11 was found in all TRAF1–3 proteins, the teleost TRAF 5 protein, and yellow catfish and chicken TRAF6 proteins.

### 2.3. Phylogenetic and Syntenic Analysis

**Figure 3:**
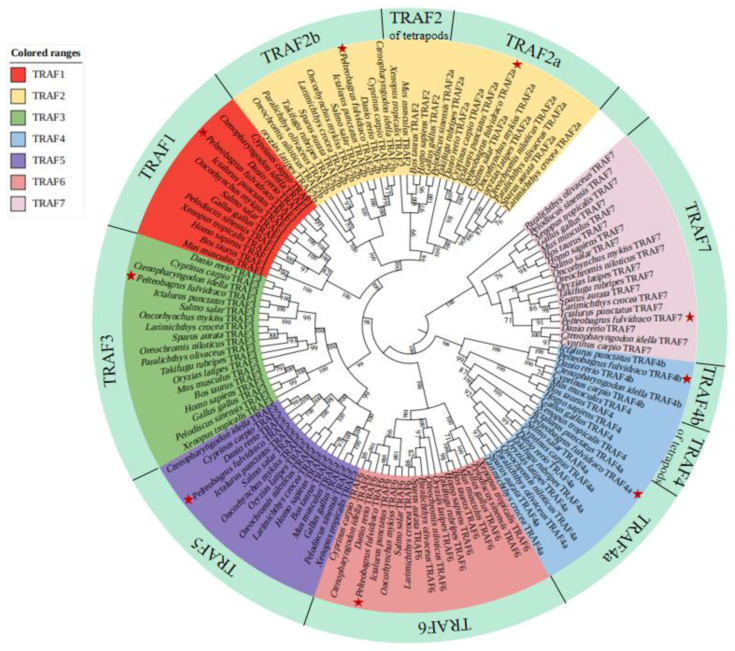
Phylogenetic relationship of TRAF genes. A neighbor-joining (NJ) phylogenetic tree was constructed using MEGA 6 with 1000 bootstrap replications based on deduced amino acid sequences of TRAF1–7 from 19 different vertebrate species: *Pelteobagrus fulvidraco*, *Homo sapiens*, *Mus musculus*, *Bos taurus*, *Gallus gallus*, *Xenopus tropicalis*, *Pelodiscus sinensis*, *Danio rerio*, *Ictalurus punctatus*, *Oncorhynchus mykiss*, *Cyprinus carpio*, *Ctenopharyngodon idella*, *Salmo salar*, *Takifugu rubripes*, *Sparus aurata*, *Oreochromis niloticus*, *Paralichthys olivaceus*, *Oryzias latipes* and *Larimichthys crocea*. The seven TRAF subfamilies are indicated in different colors. The accession numbers of the amino acid sequences of the TRAFs are provided in Supplementary Table S2.

An NJ phylogenetic tree was constructed based on deduced aa sequences of TRAF1–7 genes from 19 vertebrate species (Figure 3). The 138 aa sequences of TRAF genes were divided into seven groups (TRAF1, TRAF2, TRAF3, TRAF4, TRAF5, TRAF6 and TRAF7). The teleost TRAF2a and tetrapod TRAF2 were first clustered together, and then they clustered with teleost TRAF2b into the TRAF2 group. Afterward, the TRAF2 group clustered with the TRAF1 group into a branch. The TRAF3 and TRAF5 groups were clustered into a branch. The teleost TRAF4a and tetrapod TRAF4 were clustered together, and then they clustered with teleost TRAF4b into the TRAF4 group. Finally, the TRAF1–6 groups were clustered together into one clade, whereas the TRAF7 group was clustered as an independent clade (Figure 3).

**Figure 4:**
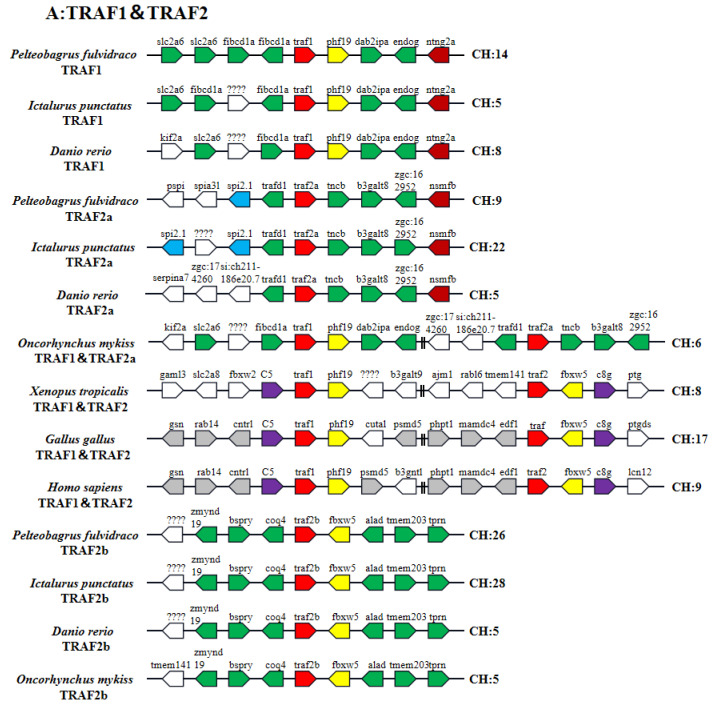

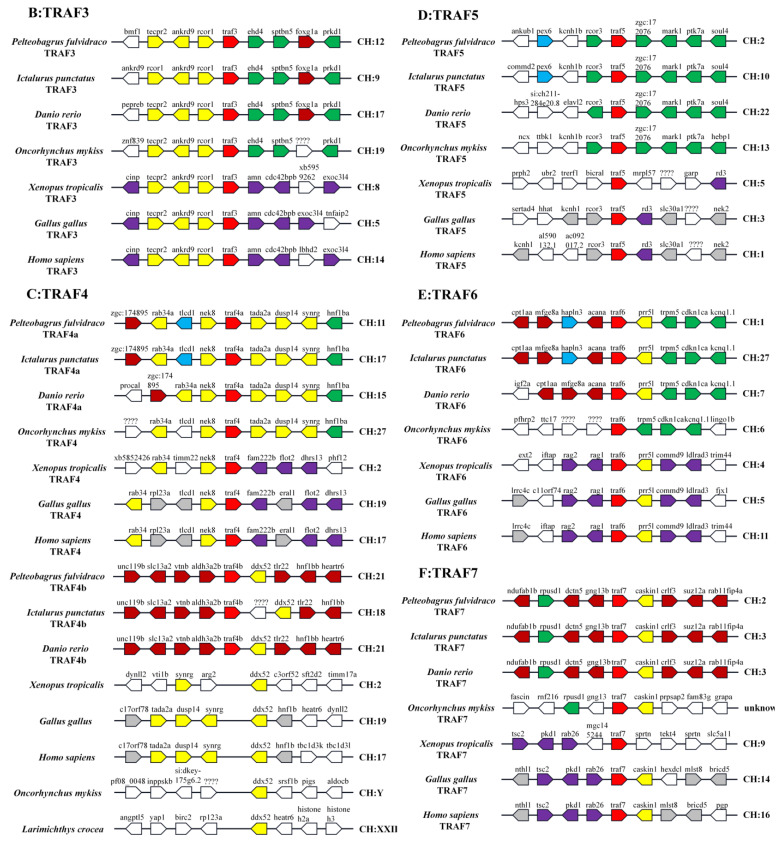
Syntenic analysis of TRAF genes. Schematic representation of gene synteny at the TRAFs in seven different vertebrate species: *Homo sapiens*, *Gallus gallus*, *Xenopus tropicalis*, *Oncorhynchus mykiss*, *Danio rerio*, *Ictalurus punctatus* and *Pelteobagrus fulvidraco*. The TRAF genes are in red, the other syntenic genes are shown to be conserved across vertebrates (yellow), only across teleosts (green), only across *X. tropicalis*, *G. gallus* and *H. sapiens* (purple), only across *P. fulvidraco* and *I. punctatus* (blue), only across *D. rerio* and *O. mykiss* (orange), only across *P. fulvidraco*, *I. punctatus* and *D. rerio* (dark red) and only across *G. gallus* and *H. sapiens* (gray).

Syntenic arrangements of TRAF family genes were shown from seven vertebrate species (Figure 4). All TRAF genes were conserved in teleosts. In African clawed frogs (*Xenopus tropicalis*), chickens and humans, TRAF1 and TRAF2 genes were located on the same chromosome. In teleosts, TRAF2 had two homologous genes (TRAF2a and TRAF2b). Unlike other teleosts, the TRAF1 and TRAF2a genes of rainbow trout and the TRAF2a and TRAF2b genes of zebra fish were located on the same chromosome, respectively. However, the surrounding gene (fbxw5) of teleost TRAF2b and tetrapod TRAF2 was highly conserved. Additionally, teleost TRAF4 also had two homologous genes (TRAF4a and TRAF4b), and the surrounding genes (nek8 and rab34) of teleost TRAF4a and tetrapod TRAF4 were highly conserved, while TRAF4b gene was absent in some fish and tetrapod genomes.

### 2.4. Tissue Expression Profiling of Pf_TRAF Genes

**Figure 5:**
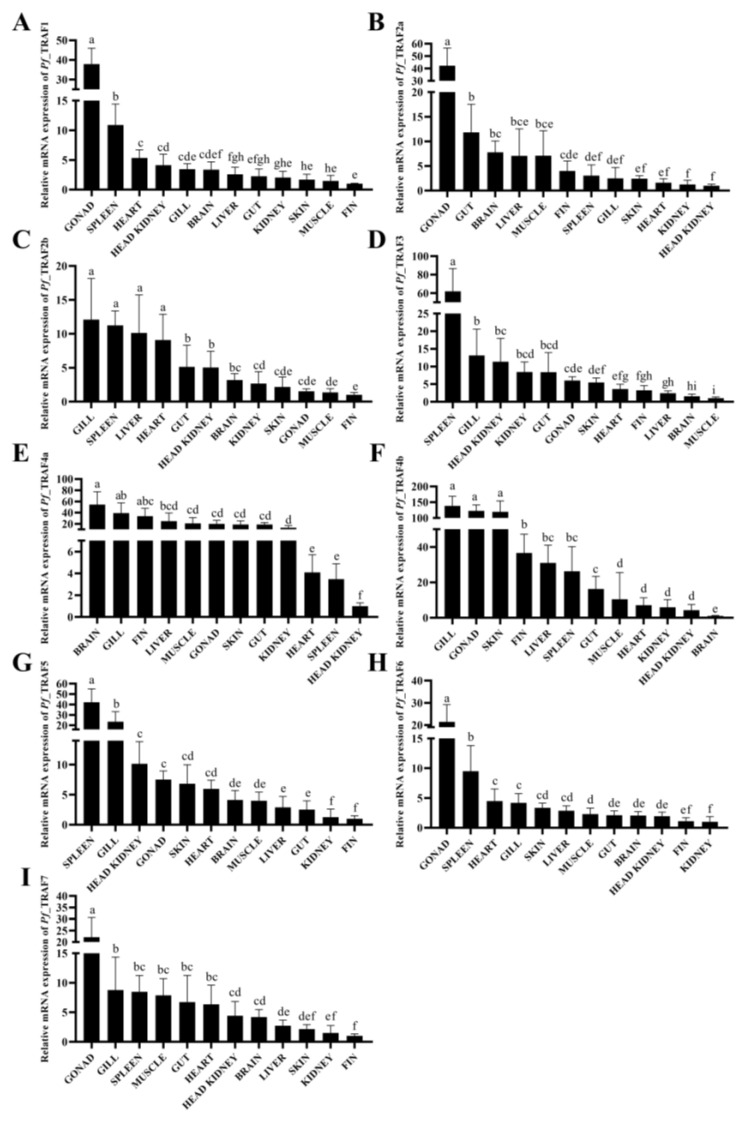
The expression distribution of *Pf_*TRAF genes in healthy yellow catfish. The mRNA expression levels of nine *Pf_*TRAF genes were examined in 12 tissues (gonad, spleen, heart, head kidney, kidney, gill, brain, liver, gut, skin, muscle and fin) of healthy yellow catfish. The β-actin gene was employed as an internal control (n = 5). Transcriptional fold changes of target gene in different tissues were calculated compared to the tissue with the lowest mRNA expression level. Different lowercase letters indicate significant differences among/between the means (one-way ANOVA and Duncan’s test, *p* < 0.05).

The mRNA expressions of nine *Pf_*TRAF genes were detected in different tissues of healthy yellow catfish by qRT-PCR. The results showed that the nine *Pf_*TRAF genes were constitutively expressed in the examined 12 tissues: gonad, spleen, heart, head kidney, kidney, gill, brain, liver, gut, muscle and fin (Figure 5). *Pf_*TRAF1, *Pf_*TRAF2a, *Pf_*TRAF4b, *Pf_*TRAF6 and *Pf_*TRAF7 mRNAs showed high expression in the gonads; *Pf_*TRAF2b, *Pf_*TRAF3 and *Pf_*TRAF5 mRNAs were found to be highly expressed in the spleen; and high expression levels of *Pf_*TRAF2b and *Pf_*TRAF4b mRNAs were observed in the gill. In addition, *Pf_*TRAF4a mRNA had a high expression level in the brain, gill, fin, liver, muscle, gonad, skin, gut and kidney. The expression levels of *Pf*_TRAF1, *Pf*_TRAF2b, *Pf*_TRAF5, *Pf*_TRAF6 and *Pf*_TRAF7 mRNAs were low in the fin; the mRNA expressions of *Pf*_TRAF2a, *Pf*_TRAF5, *Pf*_TRAF6 and *Pf*_TRAF7 were at a low level in the kidney; *Pf*_TRAF2a, *Pf*_TRAF4a and *Pf*_TRAF4b mRNAs had low expression in the head kidney; and a low expression level of *Pf*_TRAF3 mRNA was observed in the muscle and brain.

### 2.5. The Involvement of Pf_TRAF Genes in Response to E. ictaluri Infection

**Figure 6:**
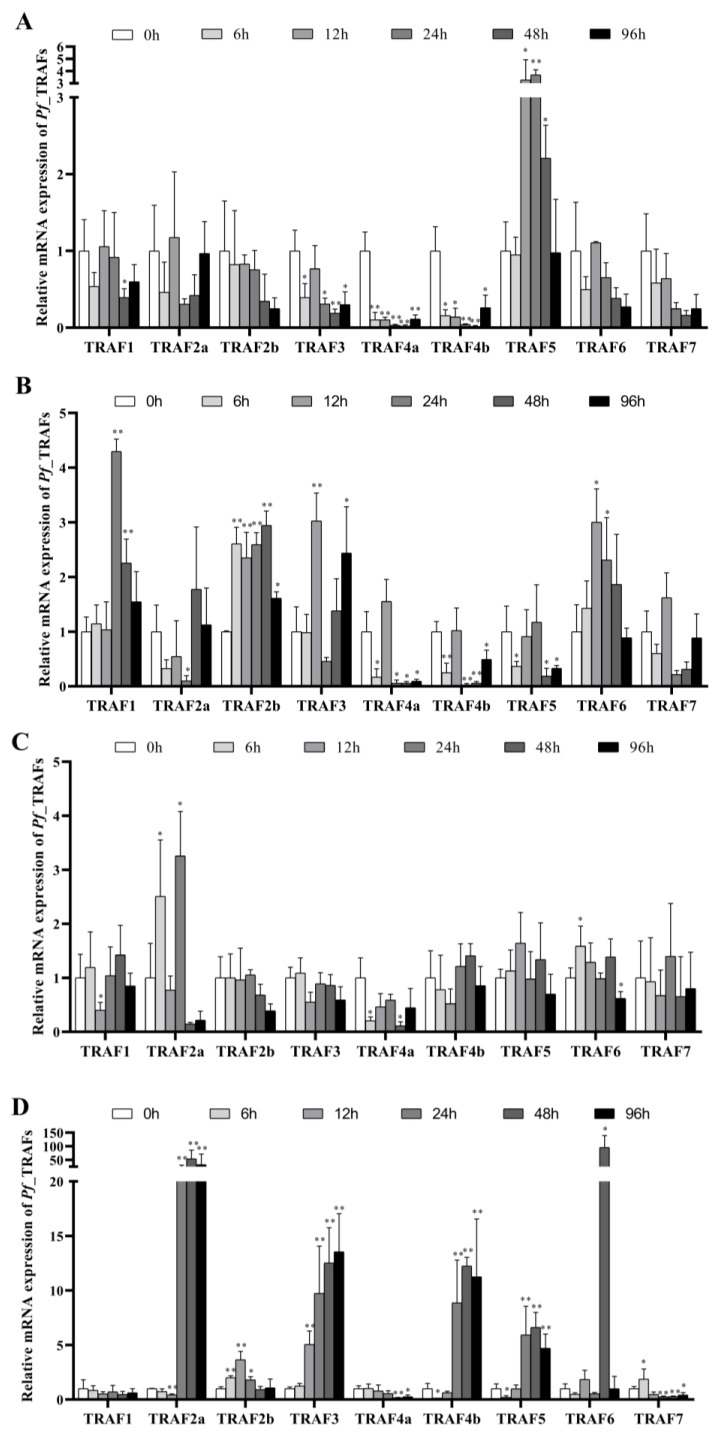
The involvement of *Pf*_TRAF genes after *E. ictaluri* infection. The mRNA expression of nine *Pf*_TRAF genes was detected in (**A**) head kidney, (**B**) spleen, (**C**) gill and (**D**) brain at different time points (0 h, 6 h, 12 h, 24 h, 48 h, 96 h) after *E. ictaluri* infection. The β-actin gene was employed as an internal control (n = 4). Transcriptional fold changes of target gene at different time points were calculated compared to the control (0 h). Significant differences at different time points after *E. ictaluri* infection compared to the control (0 h) are indicated by asterisks (*: *p* < 0.05, **: *p* < 0.01).

To investigate the role of *Pf*_TRAF genes in the immune response to pathogens, the mRNA expressions of nine *Pf*_TRAF genes in the head kidney, spleen, gill and brain of yellow catfish were examined at 0 h, 6 h, 12 h, 24 h, 48 h and 96 h after *E. ictaluri* infection (Figure 6). In the head kidney, *Pf*_TRAF5 transcripts were significantly up-regulated from 12 h to 48 h (*p* < 0.05 or 0.01), while the mRNA expressions of *Pf*_TRAF1 and *Pf*_TRAF3 were notably down-regulated at 48 h and at 6 h and 24–96 h (*p* < 0.05 or 0.01), respectively, and *Pf*_TRAF4a and *Pf*_TRAF4b mRNAs were distinctly down-regulated from 6 h to 96 h after *E. ictaluri* infection (*p* < 0.05 or 0.01) (Figure 6A). In the spleen, *Pf*_TRAF2b mRNA was significantly up-regulated from 6 h to 96 h, and the expression levels of *Pf*_TRAF1, *Pf*_TRAF3 and *Pf*_TRAF6 mRNAs were significantly up-regulated at 24–48 h, at 12 h and 96 h, and at 12–24 h, respectively, while the mRNA expressions of *Pf*_TRAF4a and *Pf*_TRAF4b were markedly down-regulated at 6 h and 24–96 h, and the *Pf*_TRAF2a and *Pf*_TRAF5 mRNAs were significantly down-regulated at certain time points after *E. ictaluri* infection (*p* < 0.05 or 0.01) (Figure 6B). In the gill, the transcripts of *Pf*_TRAF1 and *Pf*_TRAF4a genes were notably down-regulated at 12 h and at 6 h and 48 h, respectively (*p* < 0.05), while *Pf*_TRAF2a mRNA was significantly up-regulated at 6 h and 24 h, and the mRNA level of *Pf*_TRAF6 was notably up-regulated to the peak at 6 h and was significantly down-regulated at 96 h after *E. ictaluri* infection (*p* < 0.05) (Figure 6C). In the brain, the expression levels of *Pf*_TRAF2b, *Pf*_TRAF3 and *Pf*_TRAF6 mRNAs were significantly up-regulated at 6–24 h, at 12–96 h and at 48 h after *E. ictaluri* infection, respectively (*p* < 0.05 or 0.01). The mRNA levels of *Pf*_TRAF2a, *Pf*_TRAF4b and *Pf*_TRAF5 in the brain were notably down-regulated at 6 h or 12 h and then they were significantly up-regulated from 24 h to 96 h, while the expression level of *Pf*_TRAF7 mRNA was significantly up-regulated at 6 h and then significantly down-regulated from 24 h to 96 h after *E. ictaluri* infection (*p* < 0.05 or 0.01) (Figure 6D).

### 2.6. Subcellular Localization

**Figure 7:**
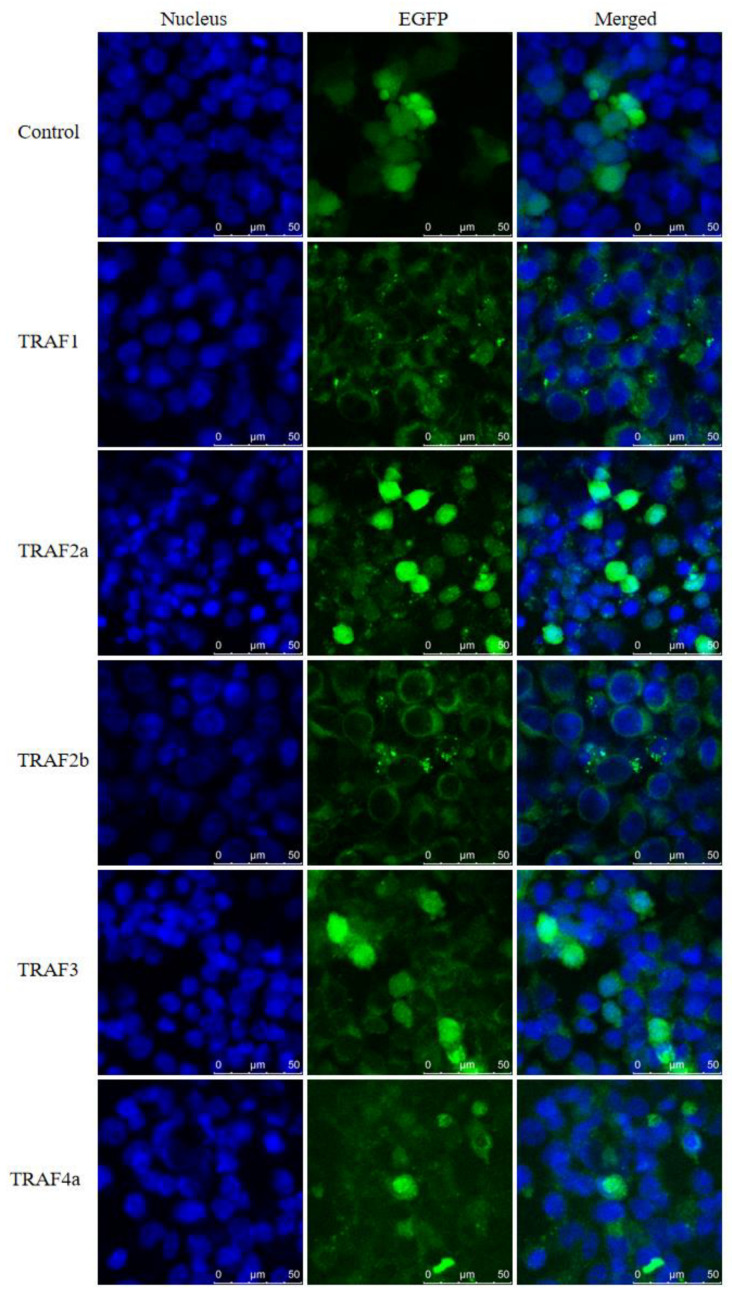

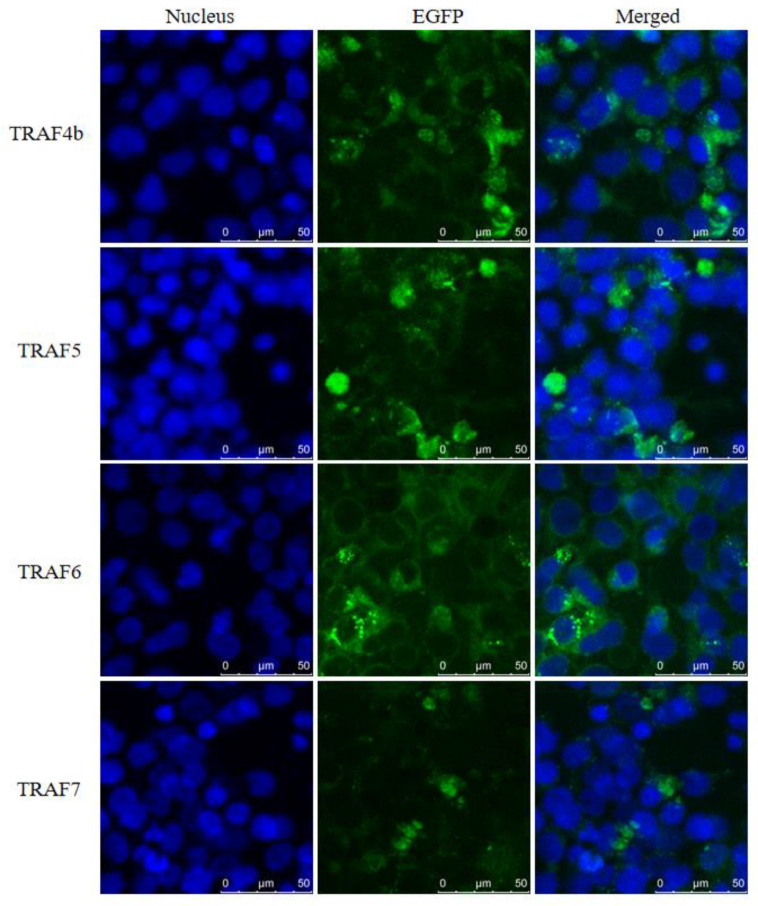
Subcellular localization of *Pf*_TRAFs in HEK293T cells. HEK293T cells were transfected with TRAFs-pEGFP-N1 plasmid or pEGFP-N1 empty plasmid (control). After cells had been cultured in 12-well plates for 36 h, the nuclei were stained with DAPI and fluorescent signals were observed under a laser confocal microscope. Scale bar: 50 μm.

To understand the cellular localization of TRAFs and to better study the structure of TRAFs protein and its potential role in cellular mechanism, the subcellular localization of *Pf_*TRAFs was determined by TRAFs-pEGFP-N1 fusion protein expression in HEK-293T cells. As shown in Figure 7, HEK293T cells were transfected with pEGFP-N1 empty plasmid or TRAFs-pEGFP-N1 plasmid, and then fluorescence signals were detected by fluorescent microscope after staining the nucleus with DAPI. The green fluorescence of EGFP could be detected in the cytoplasm and nucleus. *Pf_*TRAFs-GFP was mainly distributed in the cytoplasm, and strong fluorescence signal sites were observed in the cytoplasm of HEK293T cells. In *Pf_*TRAF2a-GFP- and *Pf_*TRAF3-GFP-transfected HEK293T cells, a strong fluorescence signal could be observed in the nucleus. *Pf_*TRAF4a-GFP was also localized in the nucleus, but the fluorescence signal was relatively weak. These results indicated that *Pf_*TRAF2a, *Pf_*TRAF3 and *Pf_*TRAF4a could be expressed in both the nucleus and the cytoplasm. Overall, *Pf_*TRAF1, *Pf_*TRAF2b, *Pf_*TRAF4b, *Pf_*TRAF5, *Pf_*TRAF6 and *Pf_*TRAF7 are mainly localized in the cytoplasm, and the other three *Pf_*TRAFs are localized in both the cytoplasm and the nucleus.

## 3. Discussion

Tumor necrosis factor receptor-associated factor (TRAF), as a class of intracellular signal transduction proteins, plays an important role in innate and adaptive immune responses by mediating NF-κB, JNK and MAPK signaling pathways [38]. The Zinc finger domain is involved in the activation of NF-κB and JNK pathways by mediating DNA binding and protein interaction [15]. The MATH domain is highly conserved and is the main domain for TRAF proteins to form homodimers or heterodimers after recruitment and bind to intracellular receptors [39,40]. The WD40 domain of TRAF7 plays a similar role to the TRAF domain in interacting with protein kinases [13]. Protein domain analysis showed that *Pf_*TRAF1–6 proteins, rather than *Pf_*TRAF7, contained a Zinc finger domain and *Pf_*TRAF1–6 proteins possessed a MATH domain, but *Pf_*TRAF7 proteins had seven continuous WD40 structures in the C-terminal region [6,9,10,11,12]. Motif composition analysis exhibited that motifs 1 and 5 were found in TRAF1–6 proteins, and motif 10 was only found in TRAF7 proteins. Phylogenetic analysis also demonstrated that TRAF1–6 groups were firstly clustered together into one branch, and TRAF7 was clustered as an independent branch. Gene structure analysis showed that vertebrate TRAF2–6 genes were highly conserved. Moreover, the gene structures of teleost TRAF1 were also highly conserved, but the gene structures of TRAF7 were different in teleosts [6,13]. Gene syntenic analysis revealed that vertebrate TRAF genes were highly conserved. These results uncovered that the function of *Pf_*TRAF1–6 proteins might be different from that of TRAF7 protein. Furthermore, teleost TRAF2 and TRAF4 have two homologous genes, and two homologous genes of *Pf_*TRAF2 or *Pf_*TRAF4 have similar protein domains. In teleosts, a third whole-genome duplication (WGD) has occurred [41]. Moreover, the common carp (*Cyprinus carpio*) and salmonid fishes had even undergone the fourth WGD [41]. The surrounding gene (fbxw5) of teleost TRAF2b and tetrapod TRAF2 was highly conserved, and the surrounding genes (nek8 and rab34) of teleost TRAF4a and tetrapod TRAF4 were highly conserved, suggesting that TRAF2 and TRAF4 homologous genes were generated by genome duplication and chromosomal rearrangement. In addition, Figure 4 shows that TRAF2a clustered first with tetrapod TRAF2 and not with fish TRAF2b and, similarly, fish TRAF4a clustered first with tetrapod TRAF4 and not with fish TRAF4b, indicating that the TRAF2b gene might be mutated more rapidly than the TRAF2a gene for some reason, resulting in the fish TRAF2a and tetrapod TRAF2 first clustering together before clustering with fish TRAF2b. A similar situation was observed for TRAF4.

TRAFs play various biological functions in life processes, such as embryonic development and innate immunity [25,38]. TRAF6 plays an important role in reproduction by maintaining ovarian development [42,43,44,45]. High levels of TRAF6 are detected in the ovary of *Xenopus laevis*, with moderate levels in the testis [46,47]. In fish, few studies have examined the TRAF expression in gonads. Lamprey (*Lampetra japonica*) TRAF3a and TRAF7a have high mRNA expressions in the ovary and testis [48]. In this study, *Pf_*TRAF1, *Pf_*TRAF2a, *Pf_*TRAF4b, *Pf_*TRAF6 and *Pf_*TRAF7 mRNAs showed high expression levels in the gonads. The spleen is an immune-related tissue, and TRAFs can be found to be highly expressed in the spleen of chicken [49], mice [50] and humans [51]. The gill is part of the mucosal tissue, which acts as the first barrier to the exposure of organisms to a complex environment by recruiting neutrophils and lymphocytes to prevent and eliminate invasive pathogens [52]. Nile tilapia TRAF6 has high mRNA expression in the spleen [53]. Chinese tongue sole TRAF4, TRAF6 and TRAF7 mRNAs are found to be highly expressed in the gill [29]. Grouper (*Epinephelus coioides*) TRAF4 and TRAF5 have high mRNA expressions in the spleen and gill, and so do the TRAF2b, TRAF3, TRAF5, TRAF6 and TRAF7 genes in black rockfish [30,54,55]. Similarly, *Pf_*TRAF2b, *Pf_*TRAF3 and *Pf_*TRAF5 mRNAs were found to be highly expressed in the spleen, and the high expression levels of *Pf_*TRAF2b, *Pf_*TRAF4a and *Pf_*TRAF4b mRNAs were observed in the gill. These results suggest that fish TRAFs may play an important role in different tissues.

In TLR, NLR and IL-17 pathways, TRAF3 and TRAF6 are important adaptor proteins to activate the TBK1/IKKε/IRF3 axis and NF-κB and MAPK pathways, respectively [48]. In large yellow croakers (*Larimichthys crocea*), TRAF3 mRNA significantly increased in the gill after *Pseudomonas plecoglossicida* stimulation [56]. Chinese tongue sole TRAF6 mRNA was up-regulated in the gill at certain time points after *V. harveyi* infection [29]. In the current study, *Pf_*TRAF3 mRNA was notably up-regulated in the spleen and brain, and *Pf_*TRAF6 mRNA was also notably up-regulated in the spleen, gill and brain of yellow catfish. TRAF2 and TRAF5 not only serve as adaptor molecules to facilitate the interaction of cIAP1/2 with RIP2 in NLR pathways, but also control mRNA stability of IL-17 as well as additional proinflammatory cytokines TNFα and IL-1β target genes in IL-17 pathways [57]. Nile tilapia TRAF5 mRNAs were significantly up-regulated at 12 h after infection with *S. agalactiae* [58]. The mRNA levels of TRAF2b and TRAF5 were significantly up-regulated in the spleen after *V. harveyi* infection in Chinese tongue sole [29]. In this study, *Pf_*TRAF2a mRNA was notably up-regulated in the gill and brain, *Pf_*TRAF2b mRNA was notably up-regulated in the spleen and brain, and *Pf_*TRAF5 mRNA was notably up-regulated in the head kidney and brain. These results imply that TRAF2a, TRAF2b, TRAF3, TRAF5 and TRAF6 may act as positive regulators in yellow catfish.

TRAF1 and TRAF4 can negatively regulate TLR and IL-17 pathways by different means [57]. In grouper, overexpressed TRAF4 also reduced the expression of interferon (IFN)-related molecules and pro-inflammatory factors [55]. In fish, it is unknown about the expression and function of TRAF1. In this study, *Pf_*TRAF1 mRNA was notably down-regulated in the head kidney and gill; *Pf_*TRAF4a mRNA was significantly down-regulated in the head kidney, spleen, gill and brain; and *Pf_*TRAF4b mRNA was significantly down-regulated in the head kidney and spleen while *Pf_*TRAF4b mRNA was significantly up-regulated in the brain. These results suggest that TRAF1, TRAF4a and TRAF4b may act as negative regulators in yellow catfish.

In mammals, little is known about the functions of TRAF7. TLR2 activates NF-κB and MKK3/6-p38 pathways via TRAF7 [25]. TRAF7 may have an agonistic activating or inhibitory effect on NF-κB pathways [13]. In lamprey, the mRNA level of TRAF7b in leukocytes was up-regulated after infections with *V. anguillarum* and *S. aureus*, and TRAF7a in leukocytes was not significantly changed [48]. In Chinese tongue sole, the mRNA levels of TRAF7 were significantly down-regulated in the gill, spleen and liver after *V. harveyi* infection [29]. In this study, after infection with *E. ictaluri*, the expression level of *Pf_*TRAF7 mRNA showed a downward trend overall in the head kidney and fluctuated in the spleen and gill, while *Pf_*TRAF7 mRNA was notably up-regulated at 6 h and then was significantly down-regulated from 12 h to 96 h in the brain. These results reveal that TRAF7 shows different immune responses to bacterial infections in different fish, and it is necessary to further study its immune function in fish such as yellow catfish.

In eukaryotic cells, the expression of a gene (i.e., transcription) always takes place in the nucleus [59]. Proteins are translated and synthesized in the cytoplasm and are directed by sorting signals to be transported to specific subcellular structures and involved in various cellular activities [60,61,62], the intracellular trafficking of proteins into organelles is a highly regulated and specific process [63], and protein functions, interactions and potential roles in the cellular mechanisms are closely related to their subcellular localization. Mature proteins must be in specific subcellular structures to perform their correct biological functions [64], and if their localization is misplaced, it will have a major impact on cellular function and even life. Therefore, understanding the location of TRAFs in cells will facilitate a better study of their protein structure and function, interactions with other proteins, and potential roles in cellular mechanisms. In mammals, all TRAF families can localize in the cytoplasm, but only TRAF4 can localize in the nucleus [65]. Compared to mammals, all six TRAFs in grouper could localize in the cytoplasm, but there was TRAF4 that could also localize in the nucleus [54,55,66,67,68]. In this study, the subcellular localization of *Pf_*TRAFs in HEK293T cells was similar to that of TRAFs in grouper. Similar to TRAF3 in grouper [59] and Nile tilapia [33], *Pf_*TRAF3 was found in both the cytoplasm and nucleus while grouper TRAF3 was localized only in cytoplasm [67]. Yellow catfish, Nile tilapia [33] and grouper [67] all have a coiled-coil domain and a MATH domain in TRAF3 while yellow catfish and Nile tilapia each have one zinc finger domain and grouper has two, suggesting that different structural features of TRAF3 may affect its subcellular localization [33,67]. In mammals, TRAF3 can inhibit both canonical and noncanonical NF-κB signaling pathways [69,70]. In HEK293T cells transfected with TRAF3 of grouper, the expression level of NF-κB was significantly up-regulated [67] and overexpression of TRAF3 in black carp and Nile tilapia also significantly activated NF-κB activity [33,71], but TRAF3 of *Lutjanus erythropterus* was found to inhibit NF-κB activation when overexpressed in M199 cells [72]. These results indicate that the regulation of NF-κB by TRAF3 in fish might be not completely consistent with that in mammals, and *Pf_*TRAF3 might play a different role in NF-κB signaling pathway. Similar to TRAF4 in mammals [73] and grouper [55], *Pf_*TRAF4a was found in both the cytoplasm and the nucleus. Yellow catfish, grouper [55] and human [73] TRAF4 proteins have a RING finger domain, three zinc finger domains and a MATH domain. It is worth noting that in human TRAF4, there is a nuclear localization signal (NLS) before the RING finger domain and in the first zinc finger domain, respectively [73]. These results suggest that *Pf*_TRAF4a might play positive regulatory roles in NF-κB signaling like other species [55,73]. The number of RING finger, zinc finger, TRAF-BIRC_3, coiled-coil and MATH domains of grouper, black carp, Nile tilapia, human and yellow catfish TRAF2 proteins is 1-2-0-1-1, 1-0-1-0-1, 1-1-1-0-1, 1-1-1-0-1 and 1-2-1-0-1, respectively [66,74,75,76]. Among them, the grouper [66] and the black carp [74] TRAF2 are localized in the cytoplasm, and the human [76] and Nile tilapia [75] TRAF2 are localized in the nucleus and cytoplasm. *Pf_*TRAF2a was also found to be present in both the nucleus and cytoplasm. It is speculated that the zinc finger domain and TRAF-BIRC_3 domain may together affect the protein subcellular localization of TRAF2, but the domain of TRAF2 protein structure controlling nuclear localization is still unclear and requires further investigation. In addition, these results also suggest that TRAF2 in yellow catfish may play a similar role to humans and Nile tilapia in negatively mediating immune signal transduction.

In summary, nine *Pf_*TRAF genes were identified in yellow catfish, and their ORF lengths were 1413–2025 bp, encoding 470–674 amino acids. Phylogenetic analysis, gene structure, conserved motifs and collinearity analysis showed that the vertebrate TRAF gene family was relatively conserved during evolution. Among them, TRAF1 is closely related to TRAF2a and TRAF2b and may have evolved from a common ancestor. TRAF7 is significantly different from other TRAFs and has a distant genetic relationship. Nine *Pf*_TRAF genes were constitutionally expressed in 12 tissues of yellow catfish, and *Pf*_TRAFs were highly expressed in immune-related tissues and gonads. The mRNA expression levels of nine *Pf*_TRAF genes were significantly changed in different tissues and at different times after infection with *E. ictaluri*, suggesting that *Pf*_TRAF genes may be involved in the immune response of yellow catfish after infection. All the nine *Pf*_TRAF genes were localized in the cytoplasm, and *Pf*_TRAF2a, *Pf*_TRAF3 and *Pf*_TRAF4a were also localized in the nucleus. Different localization results from other vertebrates suggest that *Pf*_TRAFs may play different roles in the signaling pathway. The results of this study will contribute to a better understanding of the evolution and function of TRAFs in the innate immunity of yellow catfish and other fish species.

## 4. Materials and Methods

### 4.1. Experimental Fish

The yellow catfish (one year old, ~14 g) used in this study were obtained through artificial reproduction and cultured in the circulating water tanks with continuous aeration at ~28 °C in the fish culture base of Huazhong Agricultural University (HZAU). Commercial feed (Wuxi Tongwei Feed Company, Wuxi, China) was fed twice daily (9:00 and 16:00). The animal experiments were approved by the Scientific Ethics Committee of HZAU under permit number HZAUFI-2017-013. All procedures were made with maximal efforts to minimize fish suffering.

### 4.2. Molecular Cloning and Bioinformatic Analysis

The sequences of *Pf*_TRAF gene family were downloaded from the genome database and transcriptome database of the National Center for Biotechnology Information (NCBI). Subsequently, gene-specific primers (Supplementary Table S1) were designed to identify and clone the ORF sequences of *Pf*_TRAF genes. *Pf*_TRAF genes were obtained by PCR amplification using TRAFs-ORF-F/R primers using healthy yellow catfish fin cDNA as a template, as described [77]. After assay purification, all PCR products were transformed into *Escherichia coli* competent cells and cultured as described [38]. The PCR-identified positive colonies were sequenced by Tsingke Biotech Company (Wuhan, China). The ORF sequences of the target genes were obtained using the ORF finder website (http://www.ncbi.nlm.nih.gov/projects/gorf/, accessed on 1 May 2022).

Signal peptide and protein structures were predicted using the simple modular architecture research tool (SMART) (http://smart.embl-heidelberg.de/, accessed on 5 July 2022) [78]. N-glycosylation sites were predicted using NetNGlyc (http://www.cbs.dtu.dk/services/NetNGlyc/, accessed on 5 July 2022). The theoretical isoelectric points (pI) and molecular weights of amino acids (MW) were predicted using the ExPASy (https://web.expasy.org/compute_pi/, accessed on 5 July 2022). MEGA6 software was used to construct a neighbor-joining (NJ) phylogenetic tree based on vertebrae TRAF amino acid sequences [79]. The accession numbers of vertebrate TRAF amino acid sequences and nucleotide sequences used in this study are shown in Supplementary Tables S2 and S3.

### 4.3. Tissue Collection in Healthy Fish and E. ictaluri Infected Fish

To investigate the expression pattern of *Pf_*TRAFs, 12 tissues (brain, gill, skin, liver, muscle, spleen, head kidney, gut, fin, kidney, gonad and heart) were collected from five healthy yellow catfish (one year old, ~14 g) (n = 5), and stored at −80 °C until RNA extraction.

*Edwardsiella Ictaluri* was obtained from the Laboratory of Fish Disease Prevention and Control, College of Fisheries, HZAU. Frozen *E. ictaluri* plates were streaked and inoculated onto BHI solid medium and cultured at 28 °C for 30 h. Single colonies were picked and added to 1 mL BHI liquid medium and cultured for 12 h at 28 °C with shaking. After sequencing, the bacteria were inoculated in 30 mL BHI liquid medium and cultured at 28 °C for 12 h. After centrifugation at 3000 r/min for 5 min, the bacterial solution was washed twice with 10 mL PBS (pH 7.2, c = 0.01 M), counted on a hemacyte counter plate and the bacteria were diluted to 2 × 10^7^ CFU/mL. According to the preliminary experiment, the optimal injection dose of *E. ictaluri* without fish death within five days was 1 × 10^6^ CFU per fish. Each yellow catfish in the experimental group was injected intraperitoneally with 50 μL of suspended 2 × 10^7^ CFU/mL *E. ictaluri* and each fish in the control group was injected with 50 μL of PBS (pH 7.2, c = 0.01 M). Four fish (n = 4) were sampled in the control group (0 h) and from the experimental group (n = 4) after injection of *E. ictaluri* for 6, 12, 24, 48 and 96 h, respectively. Four tissues (brain, gill, spleen and head kidney) were collected from each fish and stored at −80 °C until RNA extraction.

### 4.4. RNA Extraction, cDNA Synthesis and Quantitative Real-Time PCR Analysis

Total RNA was extracted from various tissues using Trizol Reagent (Invitrogen, Waltham, MA, USA) as described previously [41]. The first-strand cDNA was synthesized using the UEIris RT mix with DNase (US Everbright, Suzhou, China), and the cDNA products were stored at −20 °C. To detect the mRNA expression levels of *Pf_*TRAF genes in various tissues, quantitative real-time PCR (qRT-PCR) was performed using a 7300 RT-PCR system (Applied Biosystems, Waltham, MA, USA) as described previously [80]. The specific primers of *Pf_*TRAF genes for qRT-PCR were designed based on the cloned sequences of these genes (Supplementary Table S1). The β-actin gene was used as an internal reference, and the relative expression of each target gene was calculated by the 2^−ΔΔCt^ method [81].

### 4.5. Plasmid Construction

After the termination codons of *Pf_*TRAF genes (*Pf_*TRAF1, *Pf_*TRAF3, *Pf_*TRAF4a and *Pf_*TRAF6, TGA; *Pf_*TRAF2a, *Pf_*TRAF2b, *Pf_*TRAF4b and *Pf_*TRAF7, TAA; *Pf_*TRAF5, TAG) was removed by PCR amplification with specific primers (Ex-TRAFs-F/R, list in Supplementary Table S1) containing restriction sites, the PCR products and pEGFP-N1 (You Biology, Changsha, China) were digested by corresponding restriction enzymes, respectively. The digested PCR products were ligated into the pEGFP-N1 vector by T4 ligase (Takara, Dalian, China), and the ligated products were transformed into DH5α (Takara, Dalian, China) competent cells. Positive clones were selected and sequenced. The plasmid of TRAFs-pEGFP-N1 was extracted using the Endo-free Plasmid Mini Kit I (Omega Bio-tek, inc., Norcross, GA, USA).

### 4.6. Transfection of HEK293T Cells

Transfection experiments were performed using HEK293T cells stored in our laboratory. Cell slides were placed in 12-well plates, and HEK293T cells were subsequently seeded and cultured. When cells grew to 80% on the slide, they were transfected with Lipo293^TM^ transfection reagent (Beyotime, Shanghai, China). According to the instructions of transfection reagents, 50 μL DMEM culture medium was added into two centrifuge tubes, then 1 μg of TRAFs-pEGFP-N1 plasmid DNA was added into one tube and 2 μL of Lipo293^TM^ transfection reagent was added into the other tube. After gentle mixing, the culture medium containing the plasmid was added into the other tube. After standing at room temperature for 15 min, 100 μL mixture was added into the 12-well plate. The plasmid DNA of the control group was pEGFP-N1 plasmid, and other transfection procedures were the same as those of the experimental group. After 36 h of culture, the cells were washed with PBS (pH 7.4, c = 0.01 M) and fixed with 4% paraformaldehyde solution at room temperature. After washing with PBS, the cells were perforated with PBS containing 0.2% Triton-X-100 at room temperature, and then washed again and stained with DAPI (Beyotime, Shanghai, China) staining solution for 5 min at room temperature. After rinsing with PBS, the slides were sealed using a fluorescent mounting solution and observed under a laser confocal microscope.

### 4.7. Data Analyses

The data of qRT-PCRs were expressed as the mean ± standard error of the mean (SEM). The differences in mRNA expressions among various tissues of yellow catfish were examined by one-way ANOVA and Duncan’s test with STATISTICA 8.0 software. Before ANOVA analysis, all data were checked for homogeneity of variances and normality. The differences in mRNA expressions between the control group and the experimental group infected with *E. ictaluri* were compared by an independent-sample *t*-test. *p* < 0.05 was set to indicate a significant difference and *p* < 0.01 indicated a very significant difference. GraphPad Prism 5.0 software was used to plot the histograms.

## Figures and Tables

**Table 1:** The sequence information of *Pf*_TRAF genes and proteins from yellow catfish.

Gene	ORF (bp)	AA	MW (kDa)	pI
*Pf_TRAF1*	1629	542	60.55	6.95
*Pf_TRAF2a*	1839	612	69.09	7.96
*Pf_TRAF2b*	1554	517	58.76	7.90
*Pf_TRAF3*	1671	556	63.90	7.55
*Pf_TRAF4a*	1413	470	54.16	7.91
*Pf_TRAF4b*	1437	478	54.97	8.26
*Pf_TRAF5*	1653	550	62.77	6.47
*Pf_TRAF6*	1617	538	61.48	6.45
*Pf_TRAF7*	2025	674	74.86	6.64

## Data Availability

Not applicable.

## Supplementary Materials

The following supporting information can be downloaded at: https://www.mdpi.com/article/10.3390/ijms24098363/s1.
