# Supplementary material for: Molecular Characterization of Nine TRAF Genes in Yellow Catfish (Pelteobagrus fulvidraco) and Their Expression Profiling in Response to Edwardsiella ictaluri Infection
Source: Int J Mol Sci. 2023 May 6;24(9):8363. doi: 10.3390/ijms24098363 (PMC10179116; doi:10.3390/ijms24098363)
Supplement: Supplementary file 1 [file ijms-24-08363-s001.zip › ijms-2346050-supplementary.pdf]

Supplementary materials

Supplementary Table S1. Primers used in this study

Primer	Sequence (5’-3’)	Application
TRAF1-ORF-F	ATGGCTTTAGCAGCGCCTGT	ORF cloning
TRAF1-ORF-R	TCAAGATGAAGAGTCCACCACACA	ORF cloning
TRAF2a-ORF-F	ATGTTTCATTATAGCTCATGCTTT	ORF cloning
TRAF2a-ORF-R	TAAAAGGCCAGTGAGGTCAACAATG	ORF cloning
TRAF2b-ORF-F	ATGTTTCATTATAGCTCATGCTTTCT	ORF cloning
TRAF2b-ORF-R	TTAAAGGCCAGTGAGGTCAACA	ORF cloning
TRAF3-ORF-F	ATGTCCTTGCCGCACCGT	ORF cloning
TRAF3-ORF-R	TCATGGGTCAGGCAGGTCTG	ORF cloning
TRAF4a-ORF-F	ATGCCCGGCTACGACTATAAG	ORF cloning
TRAF4a-ORF-R	TCAAGCCATGATCTTTTGTGG	ORF cloning
TRAF4b-ORF-F	ATGCCTGGTTTAGATTACAAGTTTCT	ORF cloning
TRAF4b-ORF-R	TTAGTTGATTATTCTCTGCACCACAT	ORF cloning
TRAF5-ORF-F	ATGGCAGCTGAGGAGAATGAAT	ORF cloning
TRAF5-ORF-R	CTACAAATCCTCCAACCCTGTT	ORF cloning
TRAF6-ORF-F	ATGGCATCATGTAGTGACATGC	ORF cloning
TRAF6-ORF-R	TCAGAGAGATGGTTCAGGTCCTC	ORF cloning
TRAF7-ORF-F	ATGAGCTCCGGTAAATCGCACT	ORF cloning
TRAF7-ORF-R	TTAGCAGGTCCACACCTTCACG	ORF cloning
TRAF1-RT-F	ATAAACGGAGACGGAGTGGGA	qRT-PCR
TRAF1-RT-R	GGAACGATACGGACGAGAGGT	qRT-PCR
TRAF2a-RT-F	ATGGAGGCTTACAACCGC	qRT-PCR
TRAF2a-RT-R	CCTCTTCTTGGCAAAGTCCG	qRT-PCR
TRAF2b-RT-F	GTGGACATCGTTTCTGCGT	qRT-PCR
TRAF2b-RT-R	TGCTGACCCTCGTAATCTTTC	qRT-PCR
TRAF3-RT-F	AATCATTGTTCGTTTCCATCCAT	qRT-PCR
TRAF3-RT-R	ACTCGTGCTCCCTAATTTCCTG	qRT-PCR
TRAF4a-RT-F	TGCTGCCTGGCGAATACG	qRT-PCR
TRAF4a-RT-R	GGCTGCCACTGGGTTTCTG	qRT-PCR
TRAF4b-RT-F	TATGCTGGGATTCGGCTACC	qRT-PCR
TRAF4b-RT-R	GCTTTGATGAAGATGGCGTTG	qRT-PCR
TRAF5-RT-F	AAACGAAGCAAAGTGAAAGTC	qRT-PCR
TRAF5-RT-R	CTCAGCATCCAGAACATTACG	qRT-PCR
TRAF6-RT-F	TTCGGGGTCAGTTGTCGT	qRT-PCR
TRAF6-RT-R	ATGTTCGGATGGGGGTG	qRT-PCR
TRAF7-RT-F	TGGTGGTGAATAACATCGCTG	qRT-PCR
TRAF7-RT-R	GTTTGACGGTGAAAGGGCA	qRT-PCR
Ex-TRAF1-F	CCGGAATTCATGGCTTTAGCAGCGCCTGT	Plasmid construction
Ex-TRAF1-R	CGGGGTACCAGATGAAGAGTCCACCACACACT	Plasmid construction
Ex-TRAF2a-F	CCGGAATTCATGTTTCATTATAGCTCATGCTTT	Plasmid construction

Primer	Sequence (5’-3’)	Application
Ex-TRAF2a-R	CGGGGTACCAAGGCCAGTGAGGTCAACAATG	Plasmid construction
Ex-TRAF2b-F	CCGCTCGAGATGGCACGTCCATCTTTGC	Plasmid construction
Ex-TRAF2b-R	CGGGGTACCAAGTCCTGTGAGATCTACGATCGC	Plasmid construction
Ex-TRAF3-F	CCGCTCGAGATGTCCTTGCCGCACCGT	Plasmid construction
Ex-TRAF3-R	CGGGGTACCTGGGTCAGGCAGGTCTGAA	Plasmid construction
Ex-TRAF4a-F	CCGGAATTCATGCCCGGCTACGACTATAAGT	Plasmid construction
Ex-TRAF4a-R	CGGGGTACCAGCCATGATCTTTTGTGGGATC	Plasmid construction
Ex-TRAF4b-F	CCGGAATTCATGCCTGGTTTAGATTACAAGTTT	Plasmid construction
Ex-TRAF4b-R	CGGGGTACCGTTGATTATTCTCTGCACCACATC	Plasmid construction
Ex-TRAF5-F	CCGCTCGAGATGGCAGCTGAGGAGAATGAA	Plasmid construction
Ex-TRAF5-R	CGGGGTACCCAAATCCTCCAACCCTGTTGT	Plasmid construction
Ex-TRAF6-F	CCGGAATTCATGGCATCATGTAGTGACATGCAC	Plasmid construction
Ex-TRAF6-R	CGGGGTACCGAGAGATGGTTCAGGTTCTCGAGG	Plasmid construction
Ex-TRAF7-F	CCCAAGCTTATGAGCTCCGGTAAATCGCACT	Plasmid construction
Ex-TRAF7-R	CGGGGTACCGCAGGTCCACACCTTCACG	Plasmid construction
β-actin-F	TCCCTGTATGCCTCTGGTGGT	Internal reference
β-actin-R	AAGCTGTAGCCTCTCTCGGTC	Internal reference

Supplementary Table S2. List of vertebrate species and the accession numbers of their TRAF amino acid sequences used in this study

**No.**	**Species name**	**Gene name**	**Accession no.**
1	*Homo sapiens*	TRAF1	NP_005649.1
		TRAF2	NP_066961.2
		TRAF3	NP_003291.2
		TRAF4	NP_004286.2
		TRAF5	NP_001306136.1
		TRAF6	NP_665802.1
		TRAF7	NP_115647.2
2	*Mus musculus*	TRAF1	NP_033447.2
		TRAF2	NP_001277342.1
		TRAF3	NP_035762.2
		TRAF4	NP_033449.2
		TRAF5	NP_035763.2
		TRAF6	NP_033450.2
		TRAF7	NP_001165584.1
3	*Bos taurus*	TRAF1	NP_001179730.1
		TRAF2	XP_002691752.2
		TRAF3	NP_001192515.1
		TRAF4	NP_001094750.1
		TRAF5	NP_001098810.1
		TRAF6	NP_001029833.1
		TRAF7	NP_001019692.1
4	*Gallus gallus*	TRAF1	XP_024997267.1
		TRAF2	XP_015135104.1
		TRAF3	XP_040557218.1
		TRAF4	XP_015151572.3
		TRAF5	NP_001383019.1
		TRAF6	XP_004941605.1

No.	Species name	Gene name	Accession no.
		TRAF7	NP_001012546.2
5	*Xenopus tropicalis*	TRAF1	XP_031747100.1
		TRAF2	XP_031746911.1
		TRAF3	XP_031746743.1
		TRAF4	NP_001005074.1
		TRAF5	XP_004914139.2
		TRAF6	NP_001008162.2
		TRAF7	NP_001135502.1
6	*Pelodiscus sinensis*	TRAF1	XP_025039277.1
		TRAF2a	XP_006134818.1
		TRAF3	XP_025038847.1
		TRAF5	XP_006131156.1
		TRAF6	XP_006124531.1
		TRAF7	XP_014432120.1
7	*Danio rerio*	TRAF1	NP_001121853.1
		TRAF2a	XP_005172003.1
		TRAF2b	XP_005165557.1
		TRAF3	NP_001003513.1
		TRAF4a	NP_991325.1
		TRAF4b	NP_997982.1
		TRAF5	XP_021325328.1
		TRAF6	NP_001038217.1
		TRAF7	NP_001073654.1
8	*Ictalurus punctatus*	TRAF1	XP_017322461.1
		TRAF2a	XP_017307574.1
		TRAF2b	XP_017315229.1
		TRAF3	XP_017332416.1
		TRAF4a	XP_017346543.2
		TRAF4b	XP_017348734.1
		TRAF5	XP_017333882.1
		TRAF6	XP_017313922.1
		TRAF7	XP_017312723.1
9	*Oncorhynchus mykiss*	TRAF1	XP_021461029.1
		TRAF2a	XP_021462083.2
		TRAF2b	XP_021458047.1
		TRAF3	NP_001118087.1
		TRAF4	XP_021438221.1
		TRAF5	XP_021413078.1
		TRAF6	XP_036837353.1
		TRAF7	XP_036829480.1
10	*Cyprinus carpio*	TRAF1	XP_042585371.1
		TRAF2a	XP_042579944.1
		TRAF2b	XP_042580991.1
		TRAF3	XP_042598390.1
		TRAF4a	XP_042595193.1
		TRAF4b	XP_018953598.1
		TRAF5	XP_018933195.1
		TRAF6	XP_042584118.1
		TRAF7	XP_042578737.1
11	*Ctenopharyngodon idella*	TRAF1	ABE99697.1
		TRAF2b	ABE99696.1
		TRAF3	AVR54984.1
		TRAF4b	QDF63034.1
		TRAF5	QDF63035.1
		TRAF6	AGI51678.1
		TRAF7	ULE28382.1

No.	Species name	Gene name	Accession no.
12	*Salmo salar*	TRAF1	XP_045562940.1
		TRAF2a	XP_014026490.1
		TRAF2b	XP_013990611.1
		TRAF3	XP_014048262.1
		TRAF4a	NP_001167069.1
		TRAF5	XP_014026273.1
		TRAF6	XP_045564128.1
		TRAF7	XP_045571926.1
13	*Takifugu rubripes*	TRAF2a	XP_011617744.1
		TRAF2b	XP_029693846.1
		TRAF3	XP_011618072.1
		TRAF4a	XP_003968588.1
		TRAF6	XP_029702628.1
		TRAF7	XP_003964369.1
14	*Sparus aurata*	TRAF2a	XP_030273274.1
		TRAF2b	XP_030292638.1
		TRAF3	XP_030299697.1
		TRAF4a	XP_030259023.1
		TRAF6	XP_030270209.1
		TRAF7	XP_030263764.1
15	*Oreochromis niloticus*	TRAF2a	XP_025752911.1
		TRAF2b	XP_013123763.1
		TRAF3	XP_025756743.1
		TRAF4a	XP_003440320.1
		TRAF5	XP_003452807.3
		TRAF6	XP_005455785.1
		TRAF7	XP_003446520.1
16	*Paralichthys olivaceus*	TRAF2a	XP_019949664.1
		TRAF2b	XP_019937631.1
		TRAF3	XP_019962478.1
		TRAF4a	XP_019944060.1
		TRAF6	XP_019955057.1
		TRAF7	XP_019967403.1
17	*oryzias latipes*	TRAF2a	XP_011477930.1
		TRAF2b	XP_004074805.1
		TRAF3	XP_004084442.1
		TRAF4a	XP_004075368.1
		TRAF5	XP_004065654.2
		TRAF6	XP_020567582.1
		TRAF7	XP_004071899.1
18	*Larimichthys crocea*	TRAF2a	XP_019119930.1
		TRAF2b	XP_010736878.2
		TRAF3	XP_027130745.1
		TRAF4a	XP_010747668.1
		TRAF5	XP_019128363.1
		TRAF6	XP_019128980.2
		TRAF7	XP_010741701.1

Supplementary Table S3. List of vertebrate species and the accession numbers of their TRAF nucleotide sequences used in this study

No.	Species name	Gene name	Accession no.
1	*Homo sapiens*	TRAF1	NM_005658.5
		TRAF2	NM_021138.4
		TRAF3	NM_003300.4
		TRAF4	NM_004295.4
		TRAF5	NM_001319207.2
		TRAF6	NM_145803.3
		TRAF7	NM_032271.3
2	*Gallus gallus*	TRAF1	XM_025141499.2
		TRAF2	XM_015279618.3
		TRAF3	XM_040701284.2
		TRAF4	XM_015296086.4
		TRAF5	NM_001396090.1
		TRAF6	XM_004941548.5
		TRAF7	NM_001012528.3
3	*Xenopus tropicalis*	TRAF1	XM_031891240.1
		TRAF2	XM_031891051.1
		TRAF3	XM_031890883.1
		TRAF4	NM_001005074.1
		TRAF5	XM_004914082.4
		TRAF6	NM_001008161.2
		TRAF7	NM_001142030.1
4	*Danio rerio*	TRAF1	NM_001128381.1
		TRAF2a	XM_005171946.4
		TRAF2b	XM_005165500.4
		TRAF3	NM_001003513.1
		TRAF4a	NM_205762.1
		TRAF4b	NM_212817.1
		TRAF5	XM_021469653.1
		TRAF6	NM_001044752.1
		TRAF7	NM_001080185.1
5	*Ictalurus punctatus*	TRAF1	XM_017466972.3
		TRAF2a	XM_017452085.3
		TRAF2b	XM_017459740.3
		TRAF3	XM_017476927.3
		TRAF4a	XM_017491054.3
		TRAF4b	XM_017493245.3
		TRAF5	XM_017478393.3
		TRAF6	XM_017458433.3
		TRAF7	XM_017457234.3
7	*Oncorhynchus mykiss*	TRAF1	XM_021605354.2
		TRAF2a	XM_021606408.2
		TRAF2b	XM_021602372.2
		TRAF3	NM_001124615.1
		TRAF5	XM_021557403.2
		TRAF6	XM_036981458.1
		TRAF7	XM_036973585.1

Supplementary Figure S1. Nucleotide and deduced amino acid sequences of open reading frames of *Pf*_TRAFs. (A) *Pf*_TRAF1, (B) *Pf*_TRAF2a, (C) *Pf*_TRAF2b, (D) *Pf*_TRAF3, (E) *Pf*_TRAF4a, (F) *Pf*_TRAF4b, (G) *Pf*_TRAF5, (H) *Pf*_TRAF6, and (I) *Pf*_TRAF7. The start and stop codons are marked in bold. The N-glycosylation sites are marked in black circles. The RING finger domain is indicated by the black box, the zf-TRAF domain by the middle bracket, the TRAF-BIRC3-bd domain by the double arrow, the coiled-coil domain by the underline, and the WD40 domain by the curly bracket. The letters e, c, t, and h denote extended strand, random coil, β-turn, and α-helix, respectively.

A

```
1     ATGGCTTTAGCAGCGCCTGTGCCCAGCTCCCCGGGAGAGAACGAATACCCGTCCGGTTTCCCGCAGAGCATCTGTGATGAAGTTCCGGCG
1      M  A  L  A  A  P  V  P  S  S  P  G  E  N  E  Y  P  S  G  F  P  Q  S  I  C  D  E  V  P  A
       c  c  c  c  c  c  c  c  c  c  c  c  c  c  c  c  c  t  t  c  c  c  c  e  e  c  c  c  c  c
91    CAGAAATACTTGTGTACCCACTGTGACTGTGTGCTCAACGGAGCGCACCGGAGCACGTGCGGACACCGCTACTGCCTCGCGTGCGTTCAC
31     Q  K  Y  L  C  T  H  C  D  C  V  L  N  G  A  H  R  S  T  C  G  H  R  Y  C  L  A  C  V  H
       h  h  h  h  h  h  h  h  h  h  h  h  h  c  c  c  c  c  c  c  c  c  c  c  c  h  h  h  h  h
181   TGGCTCATCAGAAACAACAAAAATCTTGTGTGCACAAAGTGTAAAGAAGACCCAAACCAGCAAGGCAAGTACGGCTTACTGACTCTTGAC
61     W  L  I  R  N  N  K  N  L  V  C  T  K  C  K  E  D  P  N  Q  Q  G  K  Y  G  L  L  T  L  D
       h  h  e  c  c  c  c  c  c  c  c  c  c  c  c  c  c  c  c  c  c  c  c  h  h  h  h  h  h  h
271   CAGTTCTTCAACGACGCAGCAATCAATAAAGAGATCCTGGCTCTGAAAGTGCACTGCACCAACACTAGCTGTACGTGGAAGAGCACCTTG
91     Q  F  F  N  D  A  A  I  N  K  E  I  L  A  L  K  V  H  C  T  N  T  S  C  T  W  K  S  T  L
       h  c  c  c  h  h  h  h  h  h  h  h  h  h  h  e  e  e  c  c  c  t  t  c  c  h  h  h  h  h
361   GAGGACTTTGAGGAGTATCAGTGTCATTGTGAATACGCTTTAATCCCCTGCAACGTTGGCTGTGGGTTGATGGTTTTGAGGAAGACGTTG
121    E  D  F  E  E  Y  Q  C  H  C  E  Y  A  L  I  P  C  N  V  G  C  G  L  M  V  L  R  K  T  L
       h  h  h  h  h  c  c  c  c  c  c  e  e  e  e  c  c  c  c  c  c  c  h  h  h  h  h  h  h  h
451   GCTGCTCACCTGGACAAGGGCTGTCCCAATAATAAAAGCTTGTGTCCTACATGTTCCTGCCCACTGACCCCTTCAGAACTCCAGAAACAT
151    A  A  H  L  D  K  G  C  P  N  N  K  S  L  C  P  T  C  S  C  P  L  T  P  S  E  L  Q  K  H
       h  h  h  h  h  h  c  c  c  c  c  c  c  c  c  c  c  c  c  c  e  e  e  h  h  h  h  c  c  c
541   GTGTGTCCGAACGACAAGAAGTCCTCAAAAGCAGAGAAACGACAGAAGGATAATAAGCAAGCAAATTCCAGAAGGAAGGAGTCATGTCAG
181    V  C  P  N  D  K  K  S  S  K  A  E  K  R  Q  K  D  N  K  Q  A  N  S  R  R  K  E  S  C  Q
       c  c  c  c  c  c  c  c  c  h  h  h  h  h  h  h  c  c  c  c  c  c  c  c  c  c  c  c  c  c
631   TTCTCTGACATTGGATGCCCATTTAAGGGAACTGTAGAGAAATTGCGAGACCATGAAAGCTGCAGTTCCGCGGCCCACCTTCAGCTCCTC
211    F  S  D  I  G  C  P  F  K  G  T  V  E  K  L  R  D  H  E  S  C  S  S  A  A  H  L  Q  L  L
       e  e  e  e  c  c  c  c  c  c  h  h  h  h  h  h  h  h  h  h  h  h  h  h  h  h  h  h  h  h
721   CTGGAGGTCTTCAGAAGTGTTCGGGTCTCTGTTCCTTCCCCGCCTGAGAGGAGCGTGCACTTCCCAGGCCTGGACCTGCTCCTCCATGGC
241    L  E  V  F  R  S  V  R  V  S  V  P  S  P  P  E  R  S  V  H  F  P  G  L  D  L  L  L  H  G
       h  h  h  h  h  h  h  h  h  h  h  h  h  h  h  h  h  h  h  c  c  c  c  h  h  h  h  h  h  h
811   CAGAGCTCACCTTCTGTTTGCACTCAATCGGCTGGTGAGGTAGAGGCCGACGGGATGGATGGAGGAGTAAACGACATGGCACTCAGCCTT
271    Q  S  S  P  S  V  C  T  Q  S  A  G  E  V  E  A  D  G  M  D  G  G  V  N  D  M  A  L  S  L
       h  h  h  h  h  h  h  h  h  h  h  h  h  h  h  h  h  h  h  c  c  c  c  c  h  h  h  h  h  h
901   CATGACGACCAAGCTGAGCTGGAAGTGGGCCAGCAGCTTCACGCCCTTCAGCAGAGGCTACAGATCATGGAGAACGTCATCTCGGTACTC
301    H  D  D  Q  A  E  L  E  V  G  Q  Q  L  H  A  L  Q  Q  R  L  Q  I  M  E  N  V  I  S  V  L
       h  h  h  h  h  h  h  h  h  h  h  h  h  h  h  h  h  h  h  h  h  h  h  h  h  h  h  h  h  h
991   AGTCACGAGGTGGAGAAAACCCAGCTCACCGTGACAGCTCTGGACAGAGACACCAACACCAACATGCAGCTGGTGGAGCACTTAGACAGC
331    S  H  E  V  E  K  T  Q  L  T  V  T  A  L  D  R  D  T  N  T  N  M  Q  L  V  E  H  L  D  S
       h  h  h  h  h  h  h  h  h  h  h  h  h  h  h  h  h  h  c  c  c  h  h  h  h  h  h  h  h  h
1081  GTGGTTGCAGATCAGCAGCAGAGGATGATCAGGAAAGACCTGCACATCGCCTCGCTGCAGCAGAGCATCAGTGCGCAGCAGGACGTGTCC
361    V  V  A  D  Q  Q  Q  R  M  I  R  K  D  L  H  I  A  S  L  Q  Q  S  I  S  A  Q  Q  D  V  S
       h  h  h  h  h  h  h  h  h  h  h  h  h  h  h  h  h  h  h  h  h  h  h  h  h  h  h  h  h  c
1171  TACGACGGCACGTTTCTCTGGAAGATCTGTAATGTCAGTCAGAAGTTAAGAGAGGCAGCCACAGGGCAAAAGAGCAACCAGTATTCGCCA
391    Y  D  G  T  F  L  W  K  I  C  N  V  S  Q  K  L  R  E  A  A  T  G  Q  K  S  N  Q  Y  S  P
       c  t  t  e  e  e  e  e  h  h  h  h  h  h  h  h  h  h  h  h  t  t  c  c  c  e  e  c  c  c
1261  GCCTTCTATACATCCAGATATGGATTTAAAGTGTGCATGCGGTTGTACATAAACGGAGACGGAGTGGGAAAAGGGACGCACATGTCTCTT
421    A  F  Y  T  S  R  Y  G  F  K  V  C  M  R  L  Y  I  N  G  D  G  V  G  K  G  T  H  M  S  L
       c  e  e  c  c  c  t  t  c  e  e  e  e  e  e  e  e  t  t  c  c  c  c  c  c  c  c  e  e  e
1351  TTCTTTGTGATTATGAGAGGAGAGTATGACCCGCTTCTCTCGTGGCCTTTTAAACACAAGGTGACTTTCTTCCTCATTGACCAGAACCAA
451    F  F  V  I  M  R  G  E  Y  D  P  L  L  S  W  P  F  K  H  K  V  T  F  F  L  I  D  Q  N  Q
       e  e  e  e  e  t  c  c  t  h  h  e  e  c  c  c  c  c  c  c  e  e  e  e  e  e  c  t  t  c
1441  AGAGAGCATGTACTCGACACGTTCCGTCCCGACCTCTCGTCCGTATCGTTCCAGCGGCCCGTGTCTGACATGAACGTGGCCAGCGGCTGC
481    R  E  H  V  L  D  T  F  R  P  D  L  S  S  V  S  F  Q  R  P  V  S  D  M  N  V  A  S  G  C
       c  c  h  e  e  e  h  c  c  c  c  c  c  c  c  c  c  c  c  c  c  c  c  e  e  c  c  c  c  c
1531  CCTCTCTTCTGCCCTCTGGCCAAGCTGCGCTCCCTGAAGCATGCGTACTGCAAAGACGACACACTTTTTATCAAGTGTGTGGTGGACTCT
511    P  L  F  C  P  L  A  K  L  R  S  L  K  H  A  Y  C  K  D  D  T  L  F  I  K  C  V  V  D  S
       c  e  e  e  e  e  c  c  c  c  c  c  c  c  c  c  c  c  t  t  c  e  e  e  e  e  e  e  c  t
1621  TCATCTTGA
541    S  S  *
       t  c
```

B

```
1     ATGTTTCATTATAGCTCATGCTTTCTTTCATCCCAATGCACTGAGCTCGGTTTCATTTCCCGGCTCAGGTCACATGCTCGCTCGGGAATA
1      M F H Y S S C F L S S Q C T E L G F I S R L R S H A R S G I
       e e c c c h e e e c c c c h h h h h h h h h h h h h h h h e
91    ATAATGGCTGCACAGGAACCGTCCCCTCCGTCTTCACTGGAAGGCAACAAGCCGGGCTTCCCGAGGAAAATCCTTGCCAACAAGCTGGAA
31     I M A A Q E P S P P S S L E G N K P G F P R K I L A N K L E
       e e e c c c c c c c c c c c c c c t t c c c h h h h c c c c
181   GAAAAGCATCTGTGTAACATCTGTCAGAAAATCCTACGGAGGCCTTTTCAGGCGCAGTGCGGACACCGATTCTGTTCCTACTGCTTTAAC
61     E K H L C N I C Q K I L R R P F Q A Q C G H R F C S Y C F N
       h h h h h h h h h h h h c c c c c c c c c c c c c h h h h h
271   AAAACAGTGAGTTCTGGTCCCCAAAAATGCAGTGCTTGTATAAAAGAAGATATATTTGAGGAGCCAACATCGATTCTGAAACAGGGCTGT
91     K T V S S G P Q K C S A C I K E D I F E E P T S I L K Q G C
       h e e c c c c c c c c c c c c c c c c c c h h h h h h h c
361   GCTTTTCCTGACAACGCTGCTAAGAGAGAAGTCGAAGCCCTCGAAGCAGTTTGTATCAACGAAGGCTGCAGCTGGACAGGAACCATCAAG
121    A F P D N A A K R E V E A L E A V C I N E G C S W T G T I K
       c c c h h h h h h h h h h h e e e c c c t t c c c h e e h h
451   GAATATGAGGCTACCCACGAGGGACAGTGCGATTTTATGATCATCCCATGTCCGTCCTGCAAAGAGCTCCTGAGAGCCAACGAACTTGAA
151    E Y E A T H E G Q C D F M I I P C P S C K E L L R A N E L E
       h h h h h h c c c c c e e e e c c c c c c c h h h h h h h h
541   CGTCACAACGAGCGCGAATGCCCAGAGAGGACTCTGAACTGCAAATACTGCAAAGAACCTTTTCATTTTAAAAACATTAAGGCCCACGAT
181    R H N E R E C P E R T L N C K Y C K E P F H F K N I K A H D
       h h h h h h c c c c c c c c c c c c c c c c h h h h h h h h
631   GAGATTTGTCCAAAATACCCAATGATTTGTGAAGGCTGTGCGAAGAAGAAAATTCCCAGAGAAAAGTACGTGGATCACATTAAGCTGTGT
211    E I C P K Y P M I C E G C A K K K I P R E K Y V D H I K L C
       h c c c c c c c c c c c c c c c c c c h h h c c h h c c c c
721   ACCAAATTCCGCACCCCATGCAGATTCCACGTTGTCGGCTGCGACATGTCTGTGGAGAAGGAGAAGATCCATGAGCACGAGCGGGCATGC
241    T K F R T P C R F H V V G C D M S V E K E K I H E H E R A C
       c c c c c c c c c c e e c c c h h h h h h h h h h h h h h h
811   TCGTACGAGCACCTGAATCTGCTGCTGCACTTTATCATGGGCATCAAGGTGAGCGTAGAGAGCCTGCAGCCGCAGGGCCTGGAACTGGCC
271    S Y E H L N L L L H F I M G I K V S V E S L Q P Q G L E L A
       h h h h h h h h h h h h h h h h h h h h h h h h h h h h h h
901   AGCCACAAGCTCAAGGAGCTGCAGCAGTCTCTGTGCGAGCTGGAAGTCAAGCTGGGTCAGCTGAGCGCTGGGGCTCCCGTGCAGGGGCCC
301    S H K L K E L Q Q S L C E L E V K L G Q L S A G A P V Q G P
       h h h h h h h h h h h h h h h h h h h h h h h h c c c c c c
991   TGTGTGCCGTGCGCGCCTGCGCCTAATCCTCCACCCCCTGCGCCACCCGCACCCACCCTGACCACCTCGTTCACCCCATTGCCCAGCGCT
331    C V P C A P A P N P P P P A P P A P T L T T S F T P L P S A
       c c c c c c c c c c c c c c c c c c c h h c c c c c c c h h
1081  GTCGGCGCCGCCCTCGAGCTGCAACTGCACAGCGAGAAGACCAAGGTGGCCGAGCTGAGCCGTCGCTGCCAGGAGCTCGAGCTGAAGGCC
361    V G A A L E L Q L H S E K T K V A E L S R R C Q E L E L K A
       h h h h h h h h h h h h h h h h h h h h h h h h h h h h h h
1171  AGCACCTTCGAGAACATTGTGTGCGTGCTGAATCGCGAGATGGAGCGCTCAGCCACCACCATGGAGGCTTACAACCGCCAGCACCGGCTC
391    S T F E N I V C V L N R E M E R S A T T M E A Y N R Q H R L
       h h h h h h h h h h h h h h h h h h h h h h h h h h c c c c
1261  GACCAGGACAAGATCGAGATCCTCAACAACAAGGTACGTCAGCTGGAGAGGACGGTGGGGCTGAGGGATCTTTCCATCGTGGAGATGGAG
421    D Q D K I E I L N N K V R Q L E R T V G L R D L S I V E M E
       c h h h h h h h h h h h h h h h h h h h h h h h h h h h h h
1351  AGCAAGATGAGAGAAATGTCAGCCGCCACCTACGATGGCGTGTTTGTGTGGAGGATCACGGACTTTGCCAAGAAGAGGCAAGACGCTGTA
451    S K M R E M S A A T Y D G V F V W R I T D F A K K R Q D A V
       h h h h h h h h h c c t t e e e e e h h h h h h h h h h h h
1441  GCTGGAAGGGCCCCAGCCATGTTCTCACCTGCATTTTATACAAGCAAGTATGGCTACAAGATGTGTTTGCGCATCTACCTGAATGGGGAT
481    A G R A P A M F S P A F Y T S K Y G Y K M C L R I Y L N G D
       t t c c c e e c c c c c c c c c c c c e e e e e e e e t t t
1531  GGAACAGGGCGCGGCACTCACTTGTCTCTGTTCTTCGTGGTGATGAGGGGACACAGTGATGCCTTGCTCAAGTGGCCTTTTAACCAGAAG
511    G T G R G T H L S L F F V V M R G H S D A L L K W P F N Q K
       c c c c c c c h e e e e e e e c c c t h h h e c c c c c t t
1621  GTCACCCTGATGCTGCTCGACCAGAATAACAGAGAGCACATCATTGACGCCTTCCGGCCCGACATCTCGTCCTCGTCGTTCCAGAGACCC
541    V T L M L L D Q N N R E H I I D A F R P D I S S S S F Q R P
       e e e e e e c t t c c c h e e e e c c c c c c c c c c c c c
1711  GTCAGCGACATGAACATCGCCAGCGGATGCCCTCTCTTCTGCCCCCTCCCTAAGCTCGACTCCAAGAACTCATACATCCGTGATGACACC
571    V S D M N I A S G C P L F C P L P K L D S K N S Y I R D D T
       c c c c c c c c c c c e e e e h h h h c c c c e e e c t t c
1801  ATCTTCATCAAGGCCATTGTTGACCTCACTGGCCTTTAA
601    I F I K A I V D L T G L *
       e e e e e e e c c t t c
```

C

```
1     ATGGCACGTCCATCTTTGCCTTCTTCGATGGACTCAACGTTACCAGGTATATCCCGGCAGGTCCTGTCCGTGTCCATGGAACTTAAATAT
1      M  A  R  P  S  L  P  S  S  M  D  S  T  L  P  G  I  S  R  Q  V  L  S  V  S  M  E  L  K  Y
       c  c  c  c  c  c  c  c  c  c  c  c  c  c  t  t  c  c  c  c  e  e  e  c  c  c  c  h  h  h
91    CAGTGTCAAAAGTGCAAGGACGTTCTGAGGAAACCTTTCCAGGCGCAGTGTGGACATCGTTTCTGCGTGTTCTGTTTTAAACAACTCACC
31     Q  C  Q  K  C  K  D  V  L  R  K  P  F  Q  A  Q  C  G  H  R  F  C  V  F  C  F  K  Q  L  T
       h  h  h  h  h  h  h  h  h  c  c  c  c  c  c  c  c  c  c  c  c  c  h  h  h  h  h  h  h  h
181   AGCTCTGGCCCAATTCCTTGTGAAGCTTGTCGTGATGAAGGGATCTTTGAAGAAGAGCTCTCAATGCTCAACTTTTCTGTCGCTTTTCCA
61     S  S  G  P  I  P  C  E  A  C  R  D  E  G  I  F  E  E  E  L  S  M  L  N  F  S  V  A  F  P
       c  c  c  c  c  c  c  c  c  c  c  c  c  c  c  c  c  c  h  h  h  e  e  h  h  c  c  c  c  c
271   GACAATGCAGCACGAAGAGAAATCGACAGTTTGCCTGCCAAATGCCCTAATGACGGCTGTTCCTGGGCAGGAACTCTGAAAGATTACGAG
91     D  N  A  A  R  R  E  I  D  S  L  P  A  K  C  P  N  D  G  C  S  W  A  G  T  L  K  D  Y  E
       t  h  h  h  h  h  h  h  h  h  h  e  c  c  c  c  c  t  t  c  c  c  h  h  h  h  h  h  h  h
361   GGTCAGCACGAAGGCCGATGTGAATTCGAGCGTGTCAAGTGTGACGCGTGTCAAGTCATCATCCTTCTCAGTGAGCGAGAGCGGCACAAC
121    G  Q  H  E  G  R  C  E  F  E  R  V  K  C  D  A  C  Q  V  I  I  L  L  S  E  R  E  R  H  N
       h  h  h  h  c  c  c  c  h  e  e  e  c  c  c  c  c  c  h  h  h  h  h  h  h  h  h  h  h  h
451   GAGAGAGAGTGTGAAGCCCGTAACCTTAACTGCAAATACTGCAAAGTTTCCTTCAGCTTTAAAGATATCAAGGCTCATGATGAGATCTGT
151    E  R  E  C  E  A  R  N  L  N  C  K  Y  C  K  V  S  F  S  F  K  D  I  K  A  H  D  E  I  C
       h  h  c  c  c  c  c  h  h  c  c  c  c  c  c  c  c  c  e  h  h  h  h  h  c  c  c  c  c  c
541   TTAAAATTCCCAATGCAATGCAAAGATTGTGGTAAAAAGAAGATCCCCAGAGAAAAGTTTCAAGAGCATAGCAAATCCTGTGCCAAGTCT
181    L  K  F  P  M  Q  C  K  D  C  G  K  K  K  I  P  R  E  K  F  Q  E  H  S  K  S  C  A  K  S
       c  c  c  c  c  c  c  c  c  c  c  c  c  c  c  c  h  h  h  h  h  h  h  h  h  h  c  c  c  c
631   AAGATGGCCTGTCAGTACAGCGAGATAGGCTGCAGAGCTGTGGTGGACAACGGAAAGCAGCAGGAGCATGAACAGAGCAGTGTGATCGAG
211    K  M  A  C  Q  Y  S  E  I  G  C  R  A  V  V  D  N  G  K  Q  Q  E  H  E  Q  S  S  V  I  E
       c  c  c  c  c  c  c  c  c  c  c  c  h  h  h  h  h  h  h  h  h  h  h  h  h  h  h  h  h  h
721   CACTTGCGCCTCATGCTGGCCATGATCTCGTCGGTGCGCCCTCGACCCGAAGCAGCCGGAGAGTGGCAGGAGGACTCGGGTTTCGGCCTG
241    H  L  R  L  M  L  A  M  I  S  S  V  R  P  R  P  E  A  A  G  E  W  Q  E  D  S  G  F  G  L
       h  h  h  h  h  h  h  h  h  h  h  h  h  h  h  h  h  c  c  c  h  h  h  h  h  h  h  h  h  h
811   TACCGCGGCCCTGAAGACACGGTTCTTCCAGGCTCCCAGGGTGCATCGGGAGACTCCTGCATGCAGCGGAAGATGACGGCGCTGGAGAAC
271    Y  R  G  P  E  D  T  V  L  P  G  S  Q  G  A  S  G  D  S  C  M  Q  R  K  M  T  A  L  E  N
       h  h  h  h  h  h  h  h  h  h  h  h  h  h  h  h  h  h  h  h  h  h  h  h  h  h  h  h  h  h
901   ATCGTGTGCGTGCTGAACAGAGAGGTGGAGCGCACAGCTCTCACGCTCGAAGCTCTTGGACGACAGCACCGACTCGACCAGGAGAAGATC
301    I  V  C  V  L  N  R  E  V  E  R  T  A  L  T  L  E  A  L  G  R  Q  H  R  L  D  Q  E  K  I
       h  h  h  h  h  h  h  h  h  h  h  h  h  h  h  h  h  h  h  h  h  h  h  h  h  h  h  h  h  h
991   GAGAACCTATCCAACAAGGTGCGTCAGTTGGAGCGGACGTTAACGATGCGAGATCTGCAACTCGCCGAATCGGAACAGAATTTGCGAGAG
331    E  N  L  S  N  K  V  R  Q  L  E  R  T  L  T  M  R  D  L  Q  L  A  E  S  E  Q  N  L  R  E
       h  h  h  h  h  h  h  h  h  h  h  h  h  h  h  h  h  h  h  h  h  h  h  h  h  h  h  h  h  h
1081  CTGCAGTTTTGTACGTTCGATGGCGTGTTCATTTGGAAAATCTCCGATTTCACCCGTCGCAGACAAGATGCTCTGGCTGGACGAGCGCCT
361    L  Q  F  C  T  F  D  G  V  F  I  W  K  I  S  D  F  T  R  R  R  Q  D  A  L  A  G  R  A  P
       h  h  h  h  c  c  t  t  e  e  e  e  e  h  h  h  h  h  h  h  h  h  h  h  h  t  t  c  c  c
1171  GCCATGTTCTCGCCAGCATTCTACTCCAGTAAGTATGGCTATAAGATGTGTTTGAGGCTGTACCTGAACGGAGACGGTTCGGGAAGAAAC
391    A  M  F  S  P  A  F  Y  S  S  K  Y  G  Y  K  M  C  L  R  L  Y  L  N  G  D  G  S  G  R  N
       e  e  c  c  c  c  c  c  c  c  c  t  t  c  e  e  e  e  e  e  e  e  t  t  c  c  c  c  c  c
1261  ACTCATCTCTCGCTGTTCTTTGTGGTGATGAGAGGGAAATACGACGCTCTACTCAAATGGCCCTTCAGCCAGAAGGTAACTCTGATGCTT
421    T  H  L  S  L  F  F  V  V  M  R  G  K  Y  D  A  L  L  K  W  P  F  S  Q  K  V  T  L  M  L
       c  c  e  e  e  e  e  e  e  e  t  c  c  c  h  h  e  e  c  c  c  c     c  c  c  e  e  e  e  e
1351  CTAGACCAGAATAACCGGGAACACATCATCGATGCTTTCAGACCTGACATCAGCTCCACGTCCTTCCAACGACCCATCAGTGAGATGAAC
451    L  D  Q  N  N  R  E  H  I  I  D  A  F  R  P  D  I  S  S  T  S  F  Q  R  P  I  S  E  M  N
       e  c  t  t  c  c  c  h  e  e  e  e  c  c  c  c  c  c  c  c  c  c  c  c  c  c  c  c  c  c
1441  ATAGCCAGCGGCTGTCCACTCTTCTGTCCACTGGCCAAGCTGGCAGGAAAAAGTTCCTATGTCCGTGATGACACGATCTTTATTAAAGCG
481    I  A  S  G  C  P  L  F  C  P  L  A  K  L  A  G  K  S  S  Y  V  R  D  D  T  I  F  I  K  A
       c  c  c  c  c  c  e  e  e  e  h  h  h  h  c  c  c  c  c  e  e  c  t  t  c  e  e  e  e  e
1531  ATCGTAGATCTCACAGGACTTTAA
511    I  V  D  L  T  G  L  *
       e  e  c  t  t  t  c
```

D

```
1     ATGTCCTTGCCGCACCGTGTGGACGGGCGGGAGCCGCAGATTGCTCTGCAGCAGCAGCAGTCCTTCCTCCCGCAGCGTGGTGGTTTTCAG
1      M  S  L  P  H  R  V  D  G  R  E  P  Q  I  A  L  Q  Q  Q  Q  S  F  L  P  Q  R  G  G  F  Q
       h  c  c  c  c  c  c  c  c  c  c  c  c  c  c  c  c  c  c  c  c  c  c  c  c  c  c  c  c  c
91    GACCACTTTGTCATGCATCCTGAGCCCAAGTACTGCTGCGAGTACTGTCACATGGTGCTGTGTAAACCTCGGCAGACCGAGTGCGGACAC
31     D  H  F  V  M  H  P  E  P  K  Y  C  C  E  Y  C  H  M  V  L  C  K  P  R  Q  T  E  C  G  H
       h  e  e  e  c  c  c  c  h  h  c  c  c  h  h  h  h  e  e  e  c  c  c  c  c  c  c  c  c  c
181   CGCTTCTGCGAAAGCTGCATTATCCAGCTGCTTAGTAAAACGAATCCGGTGTGCCCAGCTGATATGGAGCCATTGTCTCCAGAAAAAATA
61     R  F  C  E  S  C  I  I  Q  L  L  S  K  T  N  P  V  C  P  A  D  M  E  P  L  S  P  E  K  I
       c  c  c  h  h  h  h  h  h  h  h  c  c  c  c  c  c  c  c  c  c  c  c  c  c  c  c  h  h  c
271   TTTCGTGATGTTTGCTGCCATAGAGAGATCATGACACTGAAAGTCTACTGCCGCAGCGCGAAAAACGGATGTAAAGAACAAATGAGTTTA
91     F  R  D  V  C  C  H  R  E  I  M  T  L  K  V  Y  C  R  S  A  K  N  G  C  K  E  Q  M  S  L
       c  c  c  h  h  h  h  h  h  h  h  h  e  e  e  e  c  c  c  c  c  c  c  c  c  c  h  h  h  h
361   CAGCAGGTTACGGACCATCTGAACATGTGTGAGTACTTTGAGGTGCCGTGTCCGTTGTGTAAAGAGAAGATTATGAGGAAGGACGTGTCT
121    Q  Q  V  T  D  H  L  N  M  C  E  Y  F  E  V  P  C  P  L  C  K  E  K  I  M  R  K  D  V  S
       h  h  h  h  h  h  h  c  c  c  c  c  e  e  e  c  c  c  c  c  c  h  h  h  h  h  h  h  h  h
451   GAACACTTGAGCCACAAATGCAAATACCGTGAGACCACCTGTGAATTCTGCAAACACAAAATGGCCTTAACGGAACTACAGAAACACAAA
151    E  H  L  S  H  K  C  K  Y  R  E  T  T  C  E  F  C  K  H  K  M  A  L  T  E  L [Q  K  H  K
       h  h  h  h  h  h  c  c  c  c  c  c  c  c  c  c  c  c  c  c  c  h  h  h  h  h  h  c  c  c
541   GAAACTGTCTGTCCTGCATTTCCAGTATCATGCCCCAATCATTGTTCGTTTCCATCCATTTTAAGAAGCGAGCTGTCCATTCACCAGCAA
181    E  T  V  C  P  A  F  P  V  S  C  P  N  H  C  S  F  P  S  I  L  R  S  E  L  S  I  H  Q  Q
       c  c  c  c  c  c  c  c  c  c  c  c  c  c  c  c  c  c  c  c  c  h  h  h  h  h  h  h  h  h
631   GATTGTCCAAAGGCTCAAGTCACATGCTCCTCCTTTCGTTTTGGCTGCAACTTCAAGGGTTTGAACCAGGAAGTTAGGGAGCGCGAGTCA
211    D  C  P  K  A  Q  V  T  C  S  S  F  R  F  G  C  N  F  K  G  L  N  Q  E  V  R  E] R  E  S
       c  c  c  c  c  c  c  c  c  c  h  h  h  h  c  c  c  h  h  h  h  h  h  h  h  h  h  h  h  h
721   AGCTTTGCCTCAGAACACTTGCGACTGATGATGGCCAGAAGCAATACTCTAGAGGCCAAGGTGGAGGACGTCAAGAGCGAGCTGCTGGAG
241    S  F  A  S  E  H  L  R  L  M  M  A  R  S  N  T  L  E  A  K  V  E  D  V  K  S  E  L  L  E
       h  h  h  h  h  h  h  h  h  h  h  h  h  h  h  h  h  h  h  h  h  h  h  h  h  h  h  h  h  h
811   CGCTACGCGGTTCTCCCGGGTCTGAGCAGTCGTCTTACAGAAGTGGAGGAACAGTACAAGGAGATGAGAGAGAAATATCGACAAGTGGAG
271    R  Y  A  V  L  P  G  L  S  S  R  L  T  E  V  E  E  Q  Y  K  E  M  R  E  K  Y  R  Q  V  E
       h  h  h  h  h  h  h  h  h  h  h  h  h  h  h  h  h  h  h  h  h  h  h  h  h  h  h  h  h  h
901   CAGAAACTCTCCAGCATGCAGAAGCTAATGAGCGCACACTCTGAGAAACTGCTGGAGGTGGAGATGGAGCTGAGGGATCTGCGGTCATTA
301    Q  K  L  S  S  M  Q  K  L  M  S  A  H  S  E  K  L  L  E  V  E  M  E  L  R  D  L  R  S  L
       h  h  h  h  h  h  h  h  h  h  h  h  h  h  h  h  h  h  h  h  h  h  h  h  h  h  h  h  h  h
991   AGGTCCATGCGGGAGGAAGTGGAAGCTCTGAGGGGTGCCGTGGAGAGCATGCGCTCTGTCGTAGCGTCGCTCGACACTGTCCGAAGCAGT
331    R  S  M  R  E  E  V  E  A  L  R  G  A  V  E  S  M  R  S  V  V  A  S  L  D  T  V  R  S  S
       h  h  h  h  h  h  h  h  h  h  h  h  h  h  h  h  h  h  h  h  h  h  h  h  h  h  h  h  c  c
1081  CCAGGTTCCCACACGCTAACCTCATTAGAGACAGAGCTGTCACGGCATGATGAGCTGCTCAGTGTGCATGACATTCGTTTGGCTGACATG
361    P  G  S  H  T  L  T  S  L  E  T  E  L  S  R  H  D  E  L  L  S  V  H  D  I  R  L  A  D  M
       c  c  h  h  h  h  h  h  h  h  h  h  h  h  h  h  h  h  h  h  h  h  h  h  h  h  h  h  h  h
1171  GACCTGAAGCTGCAGGTGCTGGAGACAGCCAGCTTCAATGGAACTCTTATTTGGAAGATTCGTGACTACAAACGGCGCAAGCAAGAAGCG
391    D  L  K  L  Q  V  L  E  T  A  S  F  N  G  T  L  I  W  K  I  R  D  Y  K  R  R  K  Q  E  A
       h  h  h  h  h  h  h  h  h  h  c  c  t  t  e  e  e  e  e  h  h  h  h  h  h  c  c  h  h  h
1261  GTGGCTTCTAAAACGTTGTCGCTTTACAGCCAGCCCTTCTATACTGGCTATTTTGGCTACAAGATGTGTGCACGTGTTTATCTGAACGGT
421    V  A  S  K  T  L  S  L  Y  S  Q  P  F  Y  T  G  Y  F  G  Y  K  M  C  A  R  V  Y  L  N  G
       h  t  t  c  c  e  e  e  e  c  c  c  c  c  c  c  c  c  c  c  e  e  e  e  e  e  e  e  t  t
1351  GACGGCATGGGGAAGGGCACCCACCTGTCGCTGTTCTTTGTGGTGATGCGTGGTGAGTATGATGCGCTGCTGCCATGGCCTTTTCGCCAA
451    D  G  M  G  K  G  T  H  L  S  L  F  F  V  V  M  R  G  E  Y  D  A  L  L  P  W  P  F  R  Q
       c  c  c  c  c  c  c  c  e  e  e  e  e  e  e  e  c  c  c  h  h  h  e  e  c  c  c  c  c  t
1441  AAAGTGACACTCATGCTAATGGACCAGGGTCCTGCACGCAAGCACTTGGGCGATGCCTTCAAGCCTGACCCAAACAGCAGCAGCTTCCGG
481    K  V  T  L  M  L  M  D  Q  G  P  A  R  K  H  L  G  D  A  F  K  P  D  P  N  S  S  S  F  R
       h  e  e  e  e  e  e  c  t  t  c  c  c  c  c  e  h  h  e  c  c  c  c  c  c  c  c  c  c  c
1531  CGGCCAACAGGGGAGATGAACATTGCATCGGGCTGTCCGCTCTTTGTCGCCCAGACCGTTCTGGAGAACGGCACCTACATCAAAGATGAC
511    R  P  T  G  E  M  N  I  A  S  G  C  P  L  F  V  A  Q  T  V  L  E  N  G  T  Y  I  K  D  D
       c  c  c  c  c  c  c  c  c  c  c  c  c  e  e  e  e  h  h  h  h  c  c  c  c  e  e  c  t  t
1621  ACCATCTTCATCAAGGTCACAGTGGACACTTCAGACCTGCCTGACCCATGA
541    T  I  F  I  K  V  T  V  D  T  S  D  L  P  D  P  *
       c  e  e  e  e  e  e  e  c  t  t  c  c  c  c  t
```

E

```
1     ATGCCCGGCTACGACTATAAGTTCCTGGAGAAGCCCAAGAGGCGCTTTCAGTGTCCGCTGTGCAGTAAAGCCATGAGAGAACCGGTGCAG
1     M P G Y D Y K F L E K P K R R F Q C P L C S K A M R E P V Q
      c t t c c e e e c c c c c c h h c c c h e e e e c c c c c e
91    GTGTCGACCTGCGGCCACAGATTCTGCGACACCTGCCTGCAGGAGTTCCTCAGTGAGGGGGTATTCAAGTGCCCCGAGGACCAGCTCCCC
31    V S T C G H R F C D T C L Q E F L S E G V F K C P E D Q L P
      e c c c c c c c c h h h h h h h h c c c c c c c c c c c c c
181   CTGGACTACGCTAAGATCTACCCTGACCCCGAGCTGGAGCAGCAGATCCTGGCTCTAGCTATCCGCTGCATCCACAGTGAGGAAGGCTGT
61    L D Y A K I Y P D P E L E Q Q I L A L A I R C I H S E E G C
      c c h h h c c c c c c h h h h h h h e e e e c c c c c c c c
271   CGCTGGACCGGCCAGATGAAGCAGCTGCAGAGCCATTTCTCTACCTGTGCATTCAATGTGATCCCGTGCCCTAACCGCTGCAGCGTCAAG
91    R W T G Q M K Q L Q S H F S T C A F N V I [P C P N R C S V K
      c h h h h h h h h h h h h c c c c c e e e c c c c c c c c h
361   CTGACACGCCGAGACCTGCCCGAGCACCTGCAGCACGACTGCCCCAAGCGCAAGGTCAAGTGCGAGTTCTGCGGCAGCGAGTTCACCGGC
121   L T R R D L P E H L Q H D C P K R K V K C E F C G S E F T G
      h h h h h h h h h h c c c c c c c c e e c c c c c c c c c h
451   GAGGCCTTCGAGAATCACCAGGGCATTTGTCCCCAGGAGAGTGTGTATTGTGAGAACAAGTGTGGAGCGAGGATGATGAGGAGACTCCTG
151   E A F E N H] [Q G I C P Q E S V Y C E N K C G A R M M R R L L
      h h h h c c c c c c c c c e e c c c c c c c c c c c h h h h
541   GCCCAGCACACCATGTCTGAGTGTCCCAAACGCACACAGCCATGCAAGTACTGCGGCAAAGAGTTTGTCTACGACACCATCCAGAATCAT
181   A Q H T M S E C P K R T Q P C K Y C G K E F V Y D T I Q N H]
      h h h h h h c c c c c c c c c c c c c c c h h h h h h h h h
631   CAGTACCACTGCCCACGTTTCCCAGTCCAGTGCCCGAACCAGTGTGGTGCCCCCAACATCGCCCGTGAAGATCTCGCAACCCATGTAAAG
211   [Q Y H C P R F P V Q C P N Q C G A P N I A R E D L A T H V K
      h c c c c c c c c c c c c c c c c c c c c h h h h h h h h h
721   GAAAGCTGCGGCAGTGCTTTAGTGCTCTGTCCCTTTAAAGAAGCTGGATGCAAGCATCGATGCCCAAAGCTGGTGATTGGCCGACACCTG
241   E S C G S A L V L C P F K E A G C K H R C P K L V I G R H] L
      h h h h h h h h h h h h h h h h c c c c c c h h h h h h h h
811   GAGGAAACCACCAAGTCCCACTTGACCATGATGTGCAACCTGGTAGGTCGTCAGCGGCAAGAGATCGCAGAGCTGCGGCGAGATCTGGAG
271   E E T T K S H L T M M C N L V G R Q R Q E I A E L R R D L E
      h h h h h h h h h h h h h h h h h h h h h h h h h h h h h h
901   GAGCTGTCAGTGAGCCATGATGGTGTACTCATCTGGAAACTGAACGATTACTCACGCAAGTTGCAGGAGGCCAAAATGCACAGCAACCAT
301   E L S V S H D G V L I W K L N D Y S R K L Q E A K M H S N H
      h h h h c c t t c e e e e h h h h h h h h h h h h c c c c c
991   GAGTTCTTCAGTCCACCCTTTTACACACACCGCTATGGCTATAAGCTGCAGGCATCCGCTTTCCTCAACGGCAACGGGAGCGGCGAAGGC
331   E F F S P P F Y T H R Y G Y K L Q A S A F L N G N G S G E G
      e e e c c c c c c c c c c c e e e e e e e e t t c c c c c c
1081  TCACATCTCTCCGTCTACATCCGTGTGCTGCCTGGCGAATACGACAACCTTCTCGAGTGGCCCTTTTCCTACAAGGTCACTTTCTCCATT
361   S H L S V Y I R V L P G E Y D N L L E W P F S Y K V T F S I
      c e e e e e e e e c t t c c h h h e c c c c c c e e e e e e
1171  ATGGACCAGAGTGATCCATCACTCTCCAAACCACAGCACATCACTGAGACGTTCAACCCGGACCCAAACTGGAAGAACTTCCAGAAACCC
391   M D Q S D P S L S K P Q H I T E T F N P D P N W K N F Q K P
      e c c c c c c c c c h h h h e e e c c c c c c c c c c c c c
1261  AGTGGCAGCCGCAGCTCACTGGACGAGAGCGCACTAGGCTTTGGCTACCCCAAGTTCGTCTCGCACGAGGAGATCCGCAAGAGGAACTAC
421   S G S R S S L D E S A L G F G Y P K F V S H E E I R K R N Y
      c c c c c c c c c c c c c c c c e e e e h h h h c c c c c c
1351  ATCCGTGACAATGCTATCTTCCTGAAGGCCTCTATTGAGATCCCACAAAAGATCATGGCTTGA
451   I R D N A I F L K A S I E I P Q K I M A *
      c c t t c e e e e e e e c c c c h e e h
```

F

```
1     ATGCCTGGTTTAGATTACAAGTTTCTGGAGCGACCGAGACGCCGCTTTCTGTGTCCTTTGTGCAGTAAACCGATGAGGGAACCTGTCCAG
1      M  P  G  L  D  Y  K  F  L  E  R  P  R  R  R  F  L  C  P  L  C  S  K  P  M  R  E  P  V  Q
       c  t  t  c  c  h  h  h  h  c  c  c  c  h  h  h  c  c  c  c  c  c  c  c  c  c  c  c  e  e
91    GTGTCCACCTGCGGACACAGGTTCTGTGACACCTGTCTGCAGGAGTTTCTCAGTGCCGGCATATTCAAGTGCCCAGAAGATCAGATGCCT
31     V  S  T  C  G  H  R  F  C  D  T  C  L  Q  E  F  L  S  A  G  I  F  K  C  P  E  D  Q  M  P
       e  e  c  c  c  c  c  c  c  h  h  h  h  h  h  h  h  h  t  c  c  c  c  c  c  c  c  c  c  c
181   CTCGACTACGCTAAAATTTTCCCAGATGTCGAGTTGGAGCAGCAGATTCTGGCTCTGCCCATCCGCTGCATTCACAGTGAGGAAGGTTGC
61     L  D  Y  A  K  I  F  P  D  V  E  L  E  Q  Q  I  L  A  L  P  I  R  C  I  H  S  E  E  G  C
       c  c  h  h  h  e  c  c  c  h  h  h  h  h  h  h  h  h  e  e  e  e  c  c  c  c  t  t  c  c
271   CGCTGGTCAGGACAGAATAAACAACTACAGGTCCATCTCTCAGTATGTACCTATAACGTGGTGCCGTGTCCAAACCGCTGCATGATGAAA
91     R  W  S  G  Q  N  K  Q  L  Q  V [H  L  S  V  C  T  Y  N  V  V  P  C  P  N  R  C  M  M  K
       c  h  h  h  h  h  h  h  h  h  h  h  h  h  t  c  c  c  e  e  e  c  c  c  c  c  c  c  h  h
361   CTGTTGCGCCGTGATCTTCCTCAGCACCTGCAGCATGACTGCACCAAGAGAAAGCTGCGCTGTGACCACTGCGCTGATGAGTTCACAGGG
121    L  L  R  R  D  L  P  Q  H  L  Q  H  D  C  T  K  R  K  L  R  C  D  H  C  A  D  E  F  T  G
       h  h  h  h  h  h  h  h  h  h  c  c  c  c  c  c  c  c  e  e  c  c  c  c  c  c  c  c  c  h
451   GAAGCACATGAGAGACACCAGGACATGTATCCAGAGGAGAGTGTGTACTGTGAGAATAAGTGTGGGGCTCGTATGGTGCGCAGGTTGCTG
151    E  A  H  E  R  H][Q  D  M  Y  P  E  E  S  V  Y  C  E  N  K  C  G  A  R  M  V  R  R  L  L
       h  h  h  h  h  c  c  c  c  c  c  c  e  e  e  e  c  c  c  t  t  c  h  h  h  h  h  h  h  h
541   ACGCAGCACAGCGTGTCCGAATGTCCTAAACGCAAACTGTCCTGCAAATACTGCAGCAAGGAGTTCCTGTACGACACCATTCAGAATCAT
181    T  Q  H  S  V  S  E  C  P  K  R  K  L  S  C  K  Y  C  S  K  E  F  L  Y  D  T  I  Q  N  H]
       h  h  h  h  h  h  h  c  c  c  c  c  c  c  c  c  c  c  c  c  c  c  h  h  h  h  h  h  h  h
631   CAGAGCCAGTGTCCACGCGTCCCAGTGCAGTGTCCTAACAGCTGTGGCACACCCGGCATCACCCGAGAGGATTTAACCATTCATGTTAAG
211   [Q  S  Q  C  P  R  V  P  V  Q  C  P  N  S  C  G  T  P  G  I  T  R  E  D  L  T  I  H  V  K
       c  c  c  c  c  c  c  c  c  c  c  c  c  c  c  c  c  c  c  c  c  h  h  h  h  h  h  h  h  h
721   GAGAGCTGTGGGACCTCCATGGTGCTCTGTCCCTTTAAGGACGCCGGCTGCAGATACAGATGCTCCAAGCCTGCAGTGTCCCTTCACCTG
241    E  S  C  G  T  S  M  V  L  C  P  F  K  D  A  G  C  R  Y  R  C  S  K  P  A  V  S  L  H] L
       t  t  c  c  c  c  e  e  e  c  c  c  c  c  t  t  c  c  c  c  c  c  c  h  h  h  h  h  h  h
811   GATGAGGCCGCTCACTCTCACCTGTCTTTACTCAGCAGCCTTGTTTCTAGGCAGAGACTCGAGCTGCAAAAGCTGAGGCGTCGTGTCGAG
271    D  E  A  A  H  S  H  L  S  L  L  S  S  L  V  S  R  Q  R  L  E  L  Q  K  L  R  R  R  V  E
       h  h  h  h  h  h  h  h  h  h  h  h  h  h  h  h  h  h  h  h  h  h  h  h  h  h  h  h  h  h
901   GAACTTTCCGGAACCCAAGACGGGACTTTACTTTGGAAAATCCCAAACTACAGCCGTCACCTACAAGAAACCAGAAATTGCTCGAATGGA
301    E  L  S  G  T  Q  D  G  T  L  L  W  K  I  P  N  Y  S  R  H  L  Q  E  T  R  N  C  S  N  G
       h  h  h  h  c  c  c  c  c  e  e  e  e  h  h  h  h  h  h  h  h  h  h  h  h  c  c  c  c  c
991   ACGTCGGAGCTCTTCAGCCCCGTTTTCTACTCTCATCGCTACGGATACCGTCTCCAAGTGTCCGTCTTTCCGAACGGAACGGCAGCGGC
331    T  S  E  L  F  S  P  V  F  Y  S  H  R  Y  G  Y  R  L  Q  V  S  V  F  P  N  G  N  G  S  G
       c  c  e  e  e  c  c  c  c  c  c  c  c  c  c  c  e  e  e  e  e  e  e  e  c  c  c  c  c  c
1081  GAAGGAACATACCTCTCCGTCTATATCAGAGTGCTCCCGGGGGAGTACGACGGCCTATTGGAGTGGCCGTTCCCTCACCGCGTCACCTTC
361    E  G  T  Y  L  S  V  Y  I  R  V  L  P  G  E  Y  D  G  L  L  E  W  P  F  P  H  R  V  T  F
       c  c  c  e  e  e  e  e  e  e  h  c  t  t  c  c  c  h  h  e  c  c  c  c  c  c  c  e  e  e
1171  TCGCTGCTGGACCAGAGCGACCCAGTACTGTCCAAACCTCAACACGTCACCGGGAGCTTCAGCCCAGATCCCAACTGGAAGAACTTCCAG
391    S  L  L  D  Q  S  D  P  V  L  S  K  P  Q  H  V  T  G  S  F  S  P  D  P  N  W  K  N  F  Q
       e  e  e  c  c  c  c  c  c  c  c  c  c  c  h  e  e  e  e  c  c  c  c  c  c  c  c  c  c  c
1261  AGACCTCGACCTGGAGCAATCGGGAGCGTACGTACCAGCCTTTTGGACGAGAGTATGCTGGGATTCGGCTACCCCAAGTTTATCTCGCAC
421    R  P  R  P  G  A  I  G  S  V  R  T  S  L  L  D  E  S  M  L  G  F  G  Y  P  K  F  I  S  H
       c  c  c  c  c  c  c  c  c  c  c  c  c  c  c  c  c  c  c  c  c  c  c  c  c  e  e  e  c  h
1351  GAGCAGATGAAGCAAAGGAACTACATCAGAGACAACGCCATCTTCATCAAAGCTTCTGTAGATGTGGTGCAGAGAATAATCAACTAA
451    E  Q  M  K  Q  R  N  Y  I  R  D  N  A  I  F  I  K  A  S  V  D  V  V  Q  R  I  I  N  *
       h  h  h  h  h  c  c  c  c  c  t  t  c  e  e  e  e  e  e  e  c  c  c  h  h  e  e  c
```

G

```
1     ATGGCAGCTGAGGAGAATGAATGCTTGAGCCTCCAGCTCTCAAAACAGAGCTCTGATCCTGTGAGGTCGTGGGAGTTGGAGTCTGTCCTG
1      M  A  A  E  E  N  E  C  L  S  L  Q  L  S  K  Q  S  S  D  P  V  R  S  W  E  L  E  S  V  L
       h  h  h  h  h  c  c  c  c  h  e  e  e  c  c  c  c  c  c  c  c  c  h  h  h  h  h  h  h  h
91    GCCAATCGCACACTCAGTTTTGTGTCCCGGCTTGAGGAGCGCTACATGTGCCCGGCCTGTGGTGACGCCATTCTTAACCCCCACCAGACG
31     A  N  R  T  L  S  F  V  S  R  L  E  E  R  Y  M  C  P  A  C  G  D  A  I  L  N  P  H  Q  T
       h  h  h  h  h  h  h  h  h  h  h  h  h  h  h  c  c  c  c  c  c  e  e  e  e  c  c  c  c  c
181   GGCTGTGGCCACATCTTCTGTGCCAAGTGCATTAGAACGTTTATTGAGAGCCGCGACACATCCAAATGTCCCTTAGATGGCATTTTGATC
61     G  C  G  H  I  F  C  A  K  C  I  R  T  F  I  E  S  R  D  T  S  K  C  P  L  D  G  I  L  I
       c  c  c  c  e  e  c  h  h  h  h  h  h  h  h  c  c  c  c  c  c  c  c  c  c  c  c  c  h  e
271   AAGTCTGAGGAGGTTTTTCAAGACAACTGCTGTAAAAGAGAACTACTAAATCTTGAAGTTTACTGTAGCAACACTCCAGATTGCACACAA
91     K  S  E  E  V  F  Q  D  N  C  C  K  R  E  L  L  N  L  E  V  Y  C  S  N  T  P  D  C  T  Q
       c  h  h  h  h  c  c  c  c  c  c  h  h  h  h  h  e  e  e  e  e  c  c  c  c  c  c  c  c  c
361   AAAGTGCCTCTGTGTAATTTGCAGAAACACCTAACTCTTTGTCAGTATGAGACCCTGCAATGCTCCAACCCCGGCTGCACTGACATCCTG
121    K  V  P  L  C  N  L  Q  K  H  L  T  L  C  Q  Y  E  T  L  Q  C  S  N  P  G  C  T  D  I  L
       h  e  e  h  h  h  h  h  h  h  h  c  c  c  c  h  h  e  e  c  c  c  c  t  t  c  c  h  h  h
451   TTACGCAAGAATGTACCAGACCACCAGCGAAACCTGTGTCAGTTTCGCACAGAGTTCTGTCACTATTGCACAAAGCCATACCCTATTACC
151    L  R  K  N  V  P  D  H  Q  R  N  L  C  Q  F  R  T  E  F  C  H  Y  C  T  K  P  Y  P  I  T
       h  h  h  h  h  h  h  h  h  h  h  c  c  c  c  c  c  c  c  c  c  c  c  c  c  c  c  c  h  h
541   CAGCTCCAGGACCATGAACAGATGTCATGCCCTGAAGCAGACGTTCAGTGTCCCAATAACTGCTCTCAGATGGTTAAGAGGCATAAGCTC
181    Q  L  Q  D  H  E  Q  M  S  C  P  E  A  D  V  Q  C  P  N  N  C  S  Q  M  V  K  R  H  K  L
       h  h  h  h  h  h  h  h  c  c  c  c  c  c  c  c  c  c  c  c  c  c  c  c  c  c  h  h  h  h
631   AAAGGACATATAGATGAGTGTCCAGAAGTGGAGACAGACTGTATTTATAAGCAATATGGCTGCACAGTCCGAGATAAACGAAGCAAAGTG
211    K  G  H  I  D  E  C  P  E  V  E  T  D  C  I  Y  K  Q  Y  G  C  T  V  R  D  K  R  S  K  V
       h  h  h  h  h  c  c  c  c  c  c  c  c  c  c  c  c  c  e  t  c  c  c  e  e  c  c  h  h  h
721   AAAGTCCATGAATACACTGAATTTAGCCATCATGTGCTACTTGTACTGGAGAGCAACAGCAAGCTTGCTAAACAGGTAGACCAGCTGCAG
241    K  V  H  E  Y  T  E  F  S  H  H  V  L  L  V  L  E  S  N  S  K  L  A  K  Q  V  D  Q  L  Q
       h  h  h  h  h  h  h  h  h  h  h  h  h  h  h  h  h  h  h  h  h  h  h  h  h  h  h  h  h  h
811   CAGGACTTGGCCCTCCAGGAGAGGGAGCTGAAAGAGAGAAACTTGCTTGTTGCTAACCTTAACAGGGAGATGACCAAGTGTGACAGCGCA
271    Q  D  L  A  L  Q  E  R  E  L  K  E  R  N  L  L  V  A  N  L  N  R  E  M  T  K  C  D  S  A
       h  h  h  h  h  h  h  h  h  h  h  h  h  h  h  h  h  h  h  h  h  h  h  h  h  h  h  h  h  h
901   TTTTCTGCAATTCAGAGGTCAGTGGTGGAGCAGAGGGAGAGCGTCTCAAGCGTTAAACAGGAGCTGCAGGAGTTGCGTAATGTTCTGGAT
301    F  S  A  I  Q  R  S  V  V  E  Q  R  E  S  V  S  S  V  K  Q  E  L  Q  E  L  R  N  V  L  D
       h  h  h  h  h  h  h  h  h  h  h  h  h  h  h  h  h  h  h  h  h  h  h  h  h  h  h  h  h  h
991   GCTGAGCTGGCTAAAGAGGAGCTGAGTGATCTCCGAGCTTCACTCGACTCTCTCAGACAGCAAGTGGCAATGACACAGAGCCTCAGAGAT
331    A  E  L  A  K  E  E  L  S  D  L  R  A  S  L  D  S  L  R  Q  Q  V  A  M  T  Q  S  L  R  D
       h  h  h  h  h  h  h  h  h  h  h  h  h  h  h  h  h  h  h  h  h  h  h  h  h  h  h  h  h  h
1081  CACCTGGGAGCTTTAGGTCAGACATGTCAGCGTCACACTCGACTACTGGACATCCATGTGGAGCAGCTGCAGTGTAACGAGCAACGTTTT
361    H  L  G  A  L  G  Q  T  C  Q  R  H  T  R  L  L  D  I  H  V  E  Q  L  Q  C  N  E  Q  R  F
       h  h  h  h  h  h  h  h  h  h  h  h  h  h  h  h  h  h  h  h  h  h  h  h  h  h  h  h  h  h
1171  CGCCAACTTGAGTCCACGTCATATGATGGAAAACTCATCTGGAAGATTCTGGACTACCAGCGTAGAAAAGACGCTGGTGCACCGCTCACC
391    R  Q  L  E  S  T  S  Y  D  G  K  L  I  W  K  I  L  D  Y  Q  R  R  K  D  A  G  A  P  L  T
       h  h  h  h  h  h  c  c  t  t  c  e  e  e  e  e  h  h  h  h  h  c  c  h  h  c  c  c  c  c
1261  TCACCGCCTTTCTACACCGGCCGCAGTGGCTACAAGCTGAGTGCCCGTGCGTACCCGGATGGGGACGGAAGCGCCCGAGGCACACACCTT
421    S  P  P  F  Y  T  G  R  S  G  Y  K  L  S  A  R  A  Y  P  D  G  D  G  S  A  R  G  T  H  L
       c  c  c  c  c  c  c  c  c  c  c  e  e  e  e  e  e  e  e  t  t  t  c  c  c  c  c  c  c  e
1351  TCTCTCTACATCATGGTGATGAGAGGTGACTTCGACTCACTGCTGCCGTGGCCATTTCGCCAGAGCGTCACACTCACCGTGCTAGACCAG
451    S  L  Y  I  M  V  M  R  G  D  F  D  S  L  L  P  W  P  F  R  Q  S  V  T  L  T  V  L  D  Q
       e  e  e  e  e  e  e  t  t  t  t  c  h  e  e  c  c  c  c  c  t  t  e  e  e  e  e  e  c  t
1441  AGTGGCTCACGCAACCACGTCAGCTGCAGCTTCACCCCTGACACCAGCAAAGAGAGCTTCCGCAGACCCACAGCTGACAGCAACGCCGCG
481    S  G  S  R  N  H  V  S  C  S  F  T  P  D  T  S  K  E  S  F  R  R  P  T  A  D  S  N  A  A
       t  c  c  c  c  c  e  e  e  e  c  c  c  c  c  c  c  c  c  c  c  c  c  c  c  c  c  c  c  c
1531  TTAGGGTTCTCTCGCTTCATCTCTCACACTGACCTGGAAGGTCCCCGAAACGCCATCTTCATACGAGATGACACTCTGTTTGTCAAGGTT
511    L  G  F  S  R  F  I  S  H  T  D  L  E  G  P  R  N  A  I  F  I  R  D  D  T  L  F  V  K  V
       t  t  c  c  e  e  e  e  h  c  c  c  c  c  c  t  t  c  e  e  e  c  t  t  c  e  e  e  e  e
1621  AAGGTTGACACAACAGGGTTGGAGGATTTGTAG
541    K  V  D  T  T  G  L  E  D  L  *
       e  e  c  c  c  c  c  c  t  h
```

H

```
1     ATGGCATCATGTAGTGACATGCACAAATCCAATCCAGATGAGTATCCATCATCCAGATGTATGGCTGATATGGAGAAGGAGTCGTATCTA
1      M  A  S  C  S  D  M  H  K  S  N  P  D  E  Y  P  S  S  R  C  M  A  D  M  E  K  E  S  Y  L
       c  c  c  c  c  h  h  c  c  c  c  c  c  c  c  c  h  h  h  h  h  h  h  h  t  t  c  c  e  e
91    AGCCCTACAGATAGCCCATCTACAATTAGTGTCTCATCCAACATCCAGGAACAGCAAGGCTATGATGTTGAATTTGATCCCCCACTGGAG
31     S  P  T  D  S  P  S  T  I  S  V  S  S  N  I  Q  E  Q  Q  G  Y  D  V  E  F  D  P  P  L  E
       c  c  c  c  c  c  c  e  e  e  c  c  c  c  c  c  c  c  c  c  c  c  e  e  e  c  c  c  c  c
181   AGCAAGTATGAATGTCCCATCTGCCTGATGGGTCTACGGTCAGCTGTGCAGACACCATGTGGTCATCGCTTCTGTAACAGTTGTATTAAA
61     S  K  Y  E [C  P  I  C  L  M  G  L  R  S  A  V  Q  T  P  C  G  H  R  F  C  N  S  C  I  K]
       c  c  c  c  c  c  e  e  e  e  e  e  c  c  c  c  c  c  c  c  c  c  c  c  c  h  h  h  h  h
271   AAATCCATCAGGGACACGGGGCAAAAATGTCCAGTTGACAATGAGGTGCTGACAGAGGACCAGCTTTTTCCAGATAACTTTGCCAAGAGG
91    [K  S  I  R  D  T  G  Q  K  C  P  V  D] N  E  V  L  T  E  D  Q  L  F  P  D  N  F  A  K  R
       h  h  h  c  c  c  c  c  c  c  c  c  c  c  c  c  c  c  c  c  c  c  c  c  c  h  h  h  h  h
361   GAAATCCTGTCTCTTACTGTACGCTGCCGCAATGTTAGCTGTAGTGACATAATGGAGCTCAGGCACCTGGAGAATCACTTGAAGCTATGT
121    E  I  L  S  L  T  V  R  C  R (N) V  S  C  S  D  I  M  E  L  R  H  L  E  N [H  L  K  L  C
       h  h  h  e  e  e  e  e  c  c  c  c  c  c  c  c  h  h  h  h  h  h  h  h  h  h  h  c  c  c
451   AAATTTGCTACAGTGCCTTGTCCCCAGTGTCAGAGGCCTGTATGGAAGAATAGTATAGAGGACCATATGAATCAGGACTGTTTACAGCGT
151    K  F  A  T  V  P  C  P  Q  C  Q  R  P  V  W  K  N  S  I  E  D  H  M  N  Q  D  C  L  Q  R
       c  e  e  e  e  c  c  c  c  c  c  c  h  h  h  h  h  h  h  h  h  h  h  h  h  h  c  c  c  c
541   CTGATCACATGCCCTGACTGTGCAGAGGGTATCATCTATGCTGACAAACAGTTTCATGAGCAGATGTGTCCCTTTGTGAATACAGTGTGT
181    L  I  T  C  P  D  C  A  E  G  I  I  Y  A  D] K  Q  F [H  E  Q  M  C  P  F  V  N  T  V  C
       c  c  c  c  c  c  c  c  c  c  c  h  h  h  h  h  h  h  h  h  c  c  c  c  c  c  c  c  c  c
631   GAGTACTGTGGAATGGATTTAATTCGGGGTCAGTTGTCGTCACACTGTGCCACAGACTGCCTGAAAGCTCCTGTTGCCTGTACGTTCAAC
211    E  Y  C  G  M  D  L  I  R  G  Q  L  S  S  H  C  A  T  D  C  L  K  A  P  V  A  C  T  F  N
       c  c  c  c  c  c  c  c  h  h  h  h  h  h  h  c  c  c  c  c  c  c  c  c  c  c  c  c  c  c
721   ATATTTGGCTGTCAAGAAAAGATGCCGAGGAACAACCTGGCCCTTCACATGCAGGAGTTTACACGGATGCACATGCGTTTCATGGCTGAC
241    I  F  G  C  Q  E  K  M  P  R  N  N  L  A  L  H] M  Q  E  F  T  R  M  H  M  R  F  M  A  D
       c  c  c  c  c  h  h  c  c  h  h  h  h  h  h  h  h  h  h  h  h  h  h  h  h  h  h  h  h  h
811   TTCCTGAGGAATCAGAGTGTGAATGGCATTCTCCCCGCTCCAAAATGTGGCCCTTCATGTTCACCCCCATCCGAACATGGTGCCAGTTCC
271    F  L  R  N  Q  S  V  N  G  I  L  P  A  P  K  C  G  P  S  C  S  P  P  S  E  H  G  A  S  S
       h  h  h  h  h  h  h  h  h  h  c  c  c  c  c  c  c  c  c  c  c  c  c  c  c  h  h  c  c  c
901   AGGGTGGGAGAGCCATGTCAGTGCAGCCAGGACATGCAGCAGTTACGTGAGACTGTTGTGGAACTTGAGGGCCGCCTCGTGCGCCAGGAC
301    R  V  G  E  P  C  Q  C  S  Q  D  M  Q  Q  L  R  E  T  V  V  E  L  E  G  R  L  V  R  Q  D
       c  c  c  c  h  h  h  h  h  h  h  h  h  h  h  h  h  h  h  h  h  h  h  h  h  h  h  h  h  h
991   CACCAGTTACGGGAGCTGAGCATTCGCAATGATACACAGCATACACAGATAACGGAGCTACGCAAGAAGCTGGCATCGCTGGAAGAGAAA
331    H  Q  L  R  E  L  S  I  R  N  D  T  Q  H  T  Q  I  T  E  L  R  K  K  L  A  S  L  E  E  K
       h  h  h  h  h  h  h  h  h  h  h  h  h  h  h  h  h  h  h  h  h  h  h  h  h  h  h  h  h  h
1081  ATGCACGAGCTGGAAGCTCAGCAGTACCAGGGTGTTTATGTATGGCGATTAGAGGGATTCTCAGCTCACCTGCAACAACAAGAAGCAGGA
361    M  H  E  L  E  A  Q  Q  Y  Q  G  V  Y  V  W  R  L  E  G  F  S  A  H  L  Q  Q  Q  E  A  G
       h  h  h  h  h  h  h  c  c  t  t  e  e  e  e  e  h  h  h  h  h  h  h  h  h  h  h  h  t  t
1171  CAACCAGTAGTGCTCCATAGCCCACCATTCTACACAGGAAAGCCAGGGTATAAGCTGTGCCTGCGACTGCACCTTCAGACACCCAGCGCG
391    Q  P  V  V  L  H  S  P  P  F  Y  T  G  K  P  G  Y  K  L  C  L  R  L  H  L  Q  T  P  S  A
       c  c  e  e  e  e  c  c  c  c  c  c  c  c  t  t  c  e  e  e  e  e  e  e  e  c  c  c  c  c
1261  CCACGTTGCTCTAACTATATCTCATTGTTTGTGCATACTATGCAAGGAGAATTTGACAGCCAGCTACCGTGGCCCCTTCAGGGCAACATC
421    P  R  C  S  N  Y  I  S  L  F  V  H  T  M  Q  G  E  F  D  S  Q  L  P  W  P  L  Q  G  N  I
       c  c  c  c  c  h  e  e  e  e  e  e  e  h  t  t  c  c  c  c  e  e  c  c  c  c  c  c  c  e
1351  CGATTGTCTGTGCTGGACCAGGCTGAGGGCCAACACCACACTGAGATAATGGAAACCAAACCAGACCTTCAGGCCTTCCAAAGGCCCACT
451    R  L  S  V  L  D  Q  A  E  G  Q  H  H  T  E  I  M  E  T  K  P  D  L  Q  A  F  Q  R  P  T
       e  e  e  e  e  c  c  c  c  c  h  h  h  h  h  h  h  c  c  c  c  c  c  c  c  c  c  c  c  c
1441  ATGCAGCGCAACCCCAAAGGCTTCGGCTATGTCACATTCATGCAGTTGCAGGCATTGCGCCAGCGTGGTTTTGTTAGGGGTGATGTGCTG
481    M  Q  R  N  P  K  G  F  G  Y  V  T  F  M  Q  L  Q  A  L  R  Q  R  G  F  V  R  G  D  V  L
       c  c  c  c  c  c  c  c  c  c  c  e  e  e  e  h  h  h  h  h  c  c  c  c  c  c  t  t  c  e
1531  CTAGTGCGCTGTGAGGTCATGCCTCGCTTTGACACAAGCCTTAGAAGAGAGGGCATGCAACCTCGAGAACCTGAACCATCTCTCTGA
511    L  V  R  C  E  V  M  P  R  F  D  T  S  L  R  R  E  G  M  Q  P  R  E  P  E  P  S  L  *
       e  e  e  e  e  c  c  c  c  c  h  h  h  c  t  t  t  c  c  c  c  c  c  c  c  c  c  c
```

I

```
1    ATGAGCTCCGGTAAATCGCACTACAACAATTTCTCAGGAGGTGTTTCATCAGCTGCAAGCATCAACGCCTGCACGTCTGACCTCAACCAC
1     M  S  S  G  K  S  H  Y  N  N  F  S  G  G  V  S  S  A  A  S  I  N  A  C  T  S  D  L  N  H
      e  c  c  c  c  c  c  c  c  c  c  c  c  c  c  c  c  c  c  c  c  c  c  c  c  c  c  c  c  c
91   AAGGACAGAATGGAGAACACGTTTGGGACGTCCTTTTCAGGAGTTCCCCCTGTTAGCAAAGCTGAGACATCCAGTAATTATAAACAGCAC
31    K  D  R  M  E  N  T  F  G  T  S  F  S  G  V  P  P  V  S  K  A  E  T  S  S  N  Y  K  Q  H
      c  c  c  h  h  h  c  c  c  c  c  c  c  c  e  e  e  e  c  c  c  c  c  c  c  c  c  c  c  c
181  CGTCGAACACCTTCAACCTCCAGCACGCTGGCTTTCTCCGCTCGAGACGATGACGACGGCATGCCACCCATCGGTACGTCACGTGGTTCA
61    R  R  T  P  S  T  S  S  T  L  A  F  S  A  R  D  D  D  D  G  M  P  P  I  G  T  S  R  G  S
      c  c  c  c  c  c  c  c  c  c  c  c  c  c  c  c  c  c  c  c  c  c  c  c  c  c  c  c  c  c
271  GACTCGGCCATCTCCGTTCGTTCTCTTCACTCGGAGTCCAACATGTCCCTGCGCTCAACCTTTTCCCTGCATGAAGAAGAGGAGGATACG
91    D  S  A  I  S  V  R  S  L  H  S  E  S  N  M  S  L  R  S  T  F  S  L  H  E  E  E  E  D  T
      c  c  h  e  e  e  c  c  c  c  c  c  c  c  c  c  c  c  c  c  c  c  c  c  c  c  c  c  c  c
361  GATCCACAGGTTTTTGCTGAACAGCCGTCAGTCAAACTGTGCTGTCAGCTGTGCTGTAACGTTTTTAAAGACCCCGTCATCACCACATGT
121   D  P  Q  V  F  A  E  Q  P  S  V  K  L  C [C  Q  L  C  C  N  V  F  K  D  P  V  I  T  T  C
      c  c  e  e  e  e  c  c  c  c  c  h  e  e  e  h  h  h  h  h  c  c  c  c  c  e  e  e  c  c
451  GGGCACACCTTCTGCAGGCGATGTGCCTTAACCTCTGAGAAATGCCCAGTGGATAACTCGAAGCTCACCGTGGTGGTGAATAACATCGCT
151   G  H  T  F  C  R  R  C  A  L  T  S  E  K] C  P  V  D  N  S  K  L  T  V  V  V  N  N  I  A
      c  c  c  e  e  h  h  e  e  e  c  c  c  c  c  c  c  c  c  c  c  e  e  e  e  e  h  h  h  h
541  GTGGCCGAGCAGATCGGTGAACTCTTCATTCACTGTAAATATGGTGTACGTCCCACTGCTTCAGGAAAACCAGCTGCCTTTGAGGTGGAT
181   V  A  E  Q  I  G  E  L  F  I  H  C  K  Y  G  V  R  P  T  A  S  G  K  P  A  A  F  E  V  D
      h  h  h  h  h  h  h  h  e  e  e  c  c  c  c  c  c  c  c  c  c  c  c  c  c  e  e  e  e  c
631  CCACTCGGCTGCCCTTTCACCGTCAAACTCAGCACTAGGAAAGATCACGAGGTGAGCTGTGATTACCGGCCGGTGCGCTGCCCGAATAAT
211   P  L  G  C  P  F  T  V  K  L  S  T  R  K  D  H  E  V  S  C  D  Y  R  P  V  R  C  P  N  N
      t  t  c  c  c  e  e  e  e  e  c  c  c  c  c  c  c  c  c  c  c  c  c  c  c  c  c  c  c  c
721  CCCAGCTGTCCTCCTCTGCTCACTATGAACCTGGAGGCTCATCTGAAGGAATGTGAACACATCAAGTGCCCCCATTCAAAATACGGATGC
241   P  S  C  P  P  L  L  T  M  N  L  E  A  H  L  K  E  C  E  H  I  K  C  P  H  S  K  Y  G  C
      c  c  c  c  c  h  h  h  h  h  h  h  h  h  h  h  h  h  h  c  c  c  c  c  c  c  c  c  c  c
811  ACGTTCATCGGGAATCAGGACACGTATGAGACGCATCTGGAAGCATGTAAGTTTGAGGGGCTGAAGGAGTTCCTGCAGCAGACAGATGAT
271   T  F  I  G  N  Q  D  T  Y  E  T  H  L  E  A  C  K  F  E  G  L  K  E  F  L  Q  Q  T  D  D
      e  e  e  c  c  h  h  h  h  h  h  h  h  h  h  h  h  h  h  h  h  h  h  h  h  h  h  h  h  h
901  CGCTTCCATGAGATGCAGGTGACTCTGGCTCAGAAAGATCAGGACATCTCCTTCCTCCGCTCCATGCTGGGCAAACTGTCCGAAAAACTC
301   R  F  H  E  M  Q  V  T  L  A  Q  K  D  Q  D  I  S  F  L  R  S  M  L  G  K  L  S  E  K  L
      h  h  h  h  h  h  h  h  h  h  h  h  h  h  h  h  h  h  h  h  h  h  h  h  h  h  h  h  h  h
991  GACCAGCTTGAGAAAAACCTGGAGCTCAAATTTGATGTGCTGGATGAGAACCAAAGCAAACTGAGTGAAGATCTTATGGAATTCCGTAGG
331   D  Q  L  E  K  N  L  E  L  K  F  D  V  L  D  E  N  Q  S  K  L  S  E  D  L  M  E  F  R  R
      h  h  h  h  h  h  h  h  h  h  h  h  h  h  h  h  h  h  h  h  h  h  h  h  h  h  h  h  h  h
1081 GACGCCTCAATGCTTAACGATGAACTTTCACATATTAACGCCAGGCTTAACATGGGCATCCTTGGCTCCTACGACCCTCAGCAGATCTTT
361   D  A  S  M  L  N  D  E  L  S  H  I  N  A  R  L  N  M  G  I  L  G  S  Y  D  P  Q  Q ┤I  F
      h  h  h  h  h  h  h  h  h  h  h  h  h  h  h  c  c  c  c  c  c  c  c  c  c  c  c  c  e  e
1171 AAGTGTAAAGGCACATTTGTGGGTCATCAGGGTCCGGTGTGGTGTTTATGTGTTTATTCCACCGGTGACCTGCTGTTCAGTGGCTCGTCA
391   K  C  K  G  T  F  V  G  H  Q  G  P  V  W  C  L  C  V  Y  S  T  G  D  L  L  F  S  G  S  S
      e  e  t  c  e  e  e  c  c  c  c  c  e  e  e  e  e  e  c  t  t  c  c  e  e  e  e  t  t  t
1261 GACAAAACCATCAAGGTTTGGGACACTTGTACCACCTACAAGTGCCAGAAAACTCTGGAGGGTCATGATGGCATTGTATTAGCACTGTGT
421   D  K  T  I  K  V  W  D├ T  C  T ┤T  Y  K  C  Q  K  T  L  E  G  H  D  G  I  V  L  A  L  C
      t  c  e  e  e  e  e  e  t  c  c  c  h  h  e  e  e  e  e  c  c  c  c  c  e  e  e  e  e  e
1351 ATTCAGGGTAATAAGCTGTACAGTGGCTCAGCAGATTGCACCATCATTGTTTGGGACATCCAGAACTTGCAGAAAGTGAACACAATCCGT
451   I  Q  G  N  K  L  Y  S  G  S  A  D  C  T  I  I  V  W  D├ I  Q ┤N  L  Q  K  V  N  T  I  R
      e  t  t  c  e  e  e  e  t  c  c  c  c  e  e  e  e  e  c  c  c  h  h  h  h  h  h  h  h  c
1441 GCACATGATAACCCAGTGTGCACACTCGTCTCCTCACACAACATGCTTTTCAGCGGCTCACTCAAAGCAATTAAGGTGTGGGACATTGTG
481   A  H  D  N  P  V  C  T  L  V  S  S  H  N  M  L  F  S  G  S  L  K  A  I  K  V  W  D├ I  V
      c  c  c  c  c  e  e  e  e  e  e  t  t  c  e  e  e  e  t  c  c  c  e  e  e  e  e  c  h  t
1531 GGCACAGAGCTGAAGCTGAAGAAAGAGCTCACGGGTCTGAATCACTGGGTTCGAGCTCTGGTCGCATCTCAGAATCACCTGTACAGCGGA
511   G  T ┤E  L  K  L  K  K  E  L  T  G  L  N  H  W  V  R  A  L  V  A  S  Q  N  H  L  Y  S  G
      t  c  h  h  h  h  h  h  h  h  h  h  h  h  h  h  h  h  h  h  e  e  t  t  c  e  e  e  e  c
1621 TCGTACCAAACCATTAAGATTTGGGACATTCGTTCTCTCGAGTGCGTACACGTCCTCCAGACCTCCGGGGGCAGCGTTTACTCCATTGCT
541   S  Y  Q  T  I  K  I  W  D├ I  R ┤S  L  E  C  V  H  V  L  Q  T  S  G  G  S  V  Y  S  I  A
      c  c  c  e  e  e  e  e  c  c  c  c  h  h  e  e  e  e  e  c  c  t  t  c  c  e  e  e  e  e
1711 GTCACCAACCACCACATAGTTTGTGGCACGTATGAGAACCTCATACATGTGTGGGACATCGAGTCTAAAGAGCAGGTGCGCACACTTACG
571   V  T  N  H  H  I  V  C  G  T  Y  E  N  L  I  H  V  W  D├ I  E ┤S  K  E  Q  V  R  T  L  T
      e  c  c  c  e  e  e  e  c  c  c  t  t  e  e  e  e  e  e  t  t  t  h  h  h  h  h  h  h  h
1801 GGACACGTAGGGACGGTGTACGCTCTGGCTGTCATTTCCACGCCGGACCAGACCAAAGTGTTCAGTGCCTCCTACGATCGCTCACTTAGG
601   G  H  V  G  T  V  Y  A  L  A  V  I  S  T  P  D  Q  T  K  V  F  S  A  S  Y  D  R  S  L  R
      t  c  c  c  h  e  e  e  e  e  e  e  c  c  c  c  c  c  e  e  e  e  c  c  t  t  c  h  e  e
1891 GTGTGGAGTATGGACAACATGATCTGCACACAGACTCTGCTGCGGCATCAAGGCAGCGTCACTGCACTGGCCGTCTCTAGAGGCCGTCTG
631   V  W  S├ M  D ┤N  M  I  C  T  Q  T  L  L  R  H  Q  G  S  V  T  A  L  A  V  S  R  G  R  L
      e  e  c  h  h  h  h  c  c  h  h  e  e  h  c  t  t  c  c  e  e  e  e  e  e  c  t  t  c  e
1981 TTTTCTGGAGCCGTGGACAGCACCGTGAAGGTGTGGACCTGCTAA
661   F  S  G  A  V  D  S  T  V  K  V  W  T├ C  *
      e  e  t  c  c  c  c  c  e  e  e  e  e  e
```

Supplementary Figure S2. Comparison of the gene organization of TRAFs genes in fish and tetrapods. Exon-intron structure of TRAFs from seven different vertebrate species: *Homo sapiens*, *Gallus gallus*, *Xenopus tropicalis, Oncorhynchus mykiss*, *Danio rerio*, *Ictalurus punctatus* and *P. fulvidraco*. The black boxes represent untranslated region, the white boxes represent exons, and the lines represent introns. The GenBank accession numbers of the TRAF nucleotide sequences are listed in Supplementary Table S3.

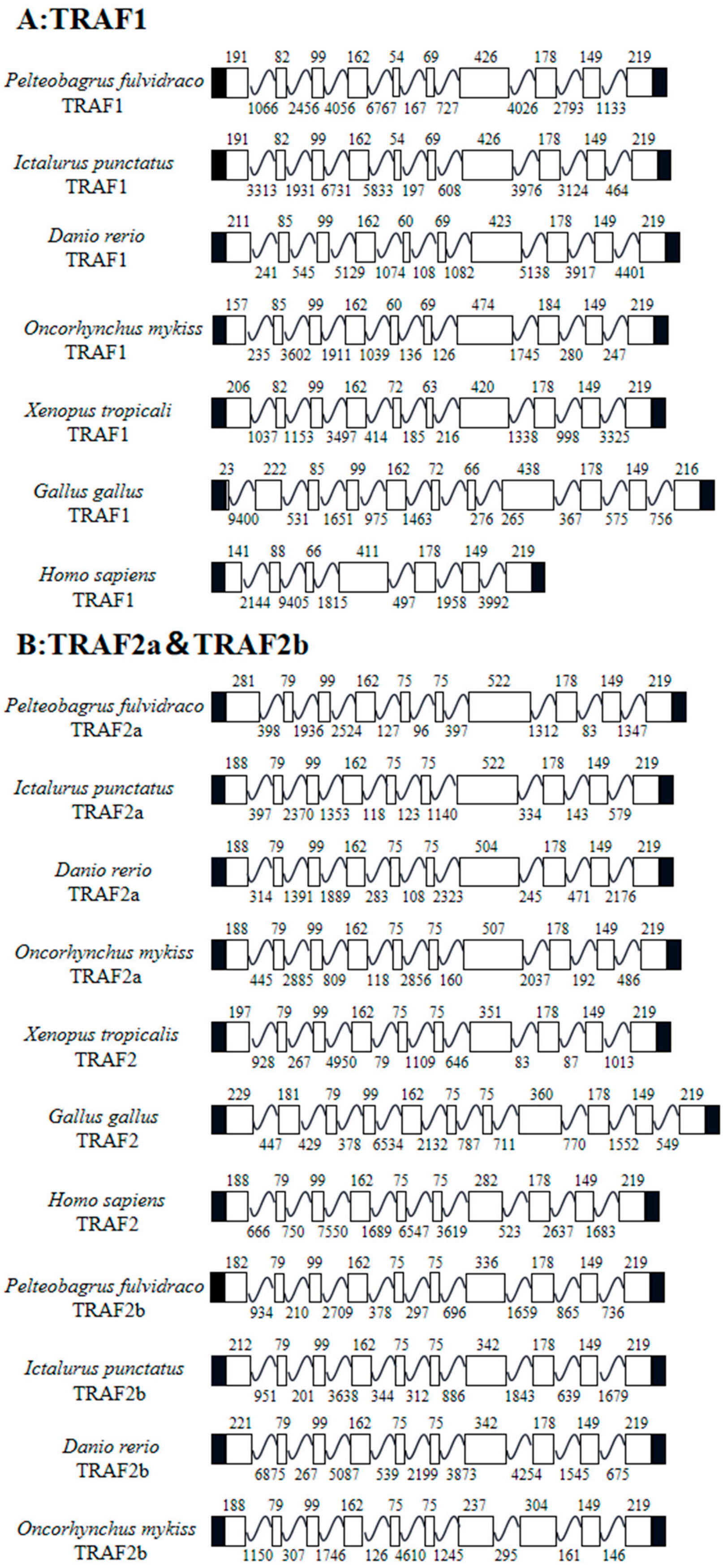
A:TRAF1
191 82 99 162 54 69 426 178 149 219
Pelteobagrus fulvidraco TRAF1
1066 2456 4056 6767 167 727 4026 2793 1133
191 82 99 162 54 69 426 178 149 219
Ictalurus punctatus TRAF1
3313 1931 6731 5833 197 608 3976 3124 464
211 85 99 162 60 69 423 178 149 219
Danio rerio TRAF1
241 545 5129 1074 108 1082 5138 3917 4401
157 85 99 162 60 69 474 184 149 219
Oncorhynchus mykiss TRAF1
235 3602 1911 1039 136 126 1745 280 247
206 82 99 162 72 63 420 178 149 219
Xenopus tropicali TRAF1
1037 1153 3497 414 185 216 1338 998 3325
23 222 85 99 162 72 66 438 178 149 216
Gallus gallus TRAF1
9400 531 1651 975 1463 276 265 367 575 756
141 88 66 411 178 149 219
Homo sapiens TRAF1
2144 9405 1815 497 1958 3992
B:TRAF2a＆TRAF2b
281 79 99 162 75 75 522 178 149 219
Pelteobagrus fulvidraco TRAF2a
398 1936 2524 127 96 397 1312 83 1347
188 79 99 162 75 75 522 178 149 219
Ictalurus punctatus TRAF2a
397 2370 1353 118 123 1140 334 143 579
188 79 99 162 75 75 504 178 149 219
Danio rerio TRAF2a
314 1391 1889 283 108 2323 245 471 2176
188 79 99 162 75 75 507 178 149 219
Oncorhynchus mykiss TRAF2a
445 2885 809 118 2856 160 2037 192 486
197 79 99 162 75 75 351 178 149 219
Xenopus tropicalis TRAF2
928 267 4950 79 1109 646 83 87 1013
229 181 79 99 162 75 75 360 178 149 219
Gallus gallus TRAF2
447 429 378 6534 2132 787 711 770 1552 549
188 79 99 162 75 75 282 178 149 219
Homo sapiens TRAF2
666 750 7550 1689 6547 3619 523 2637 1683
182 79 99 162 75 75 336 178 149 219
Pelteobagrus fulvidraco TRAF2b
934 210 2709 378 297 696 1659 865 736
212 79 99 162 75 75 342 178 149 219
Ictalurus punctatus TRAF2b
951 201 3638 344 312 886 1843 639 1679
221 79 99 162 75 75 342 178 149 219
Danio rerio TRAF2b
6875 267 5087 539 2199 3873 4254 1545 675
188 79 99 162 75 75 237 304 149 219
Oncorhynchus mykiss TRAF2b
1150 307 1746 126 4610 1245 295 161 146

C:TRAF3

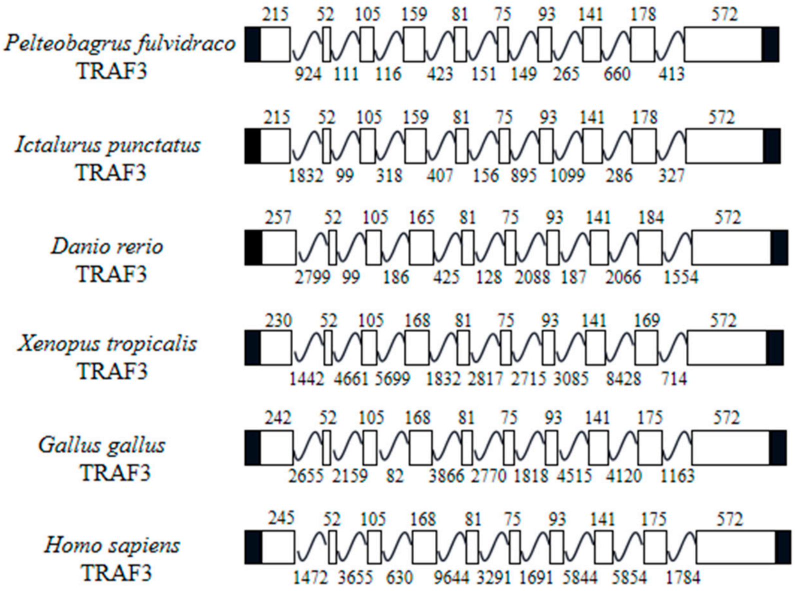

D:TRAF4a&TRAF4b

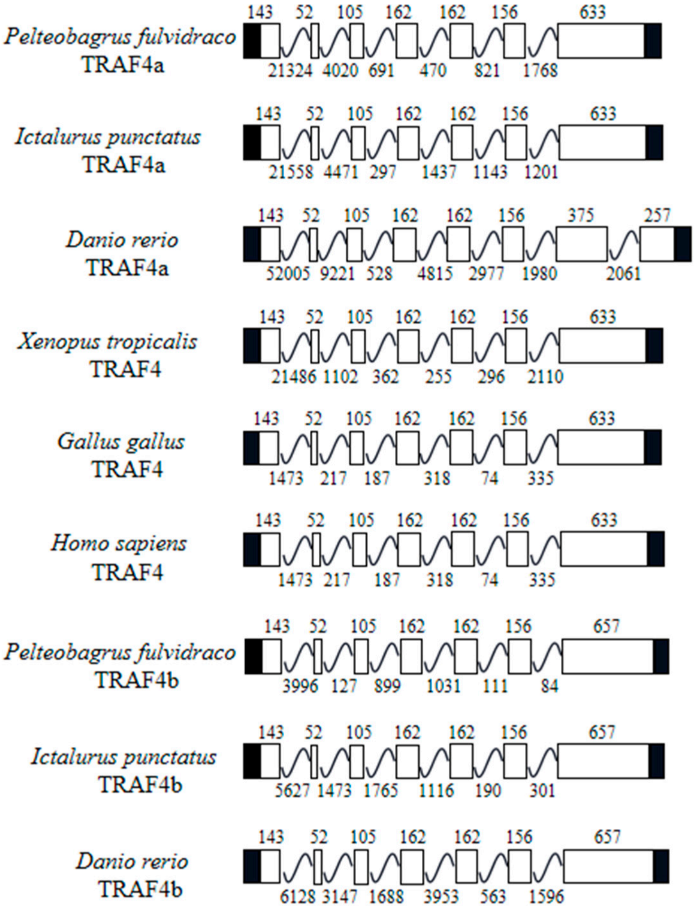

E:TRAF5

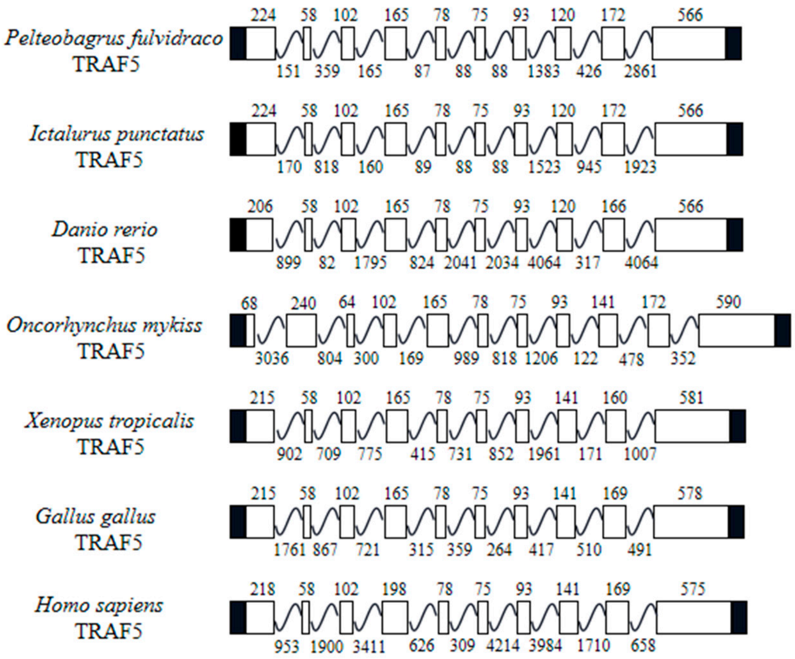

F:TRAF6

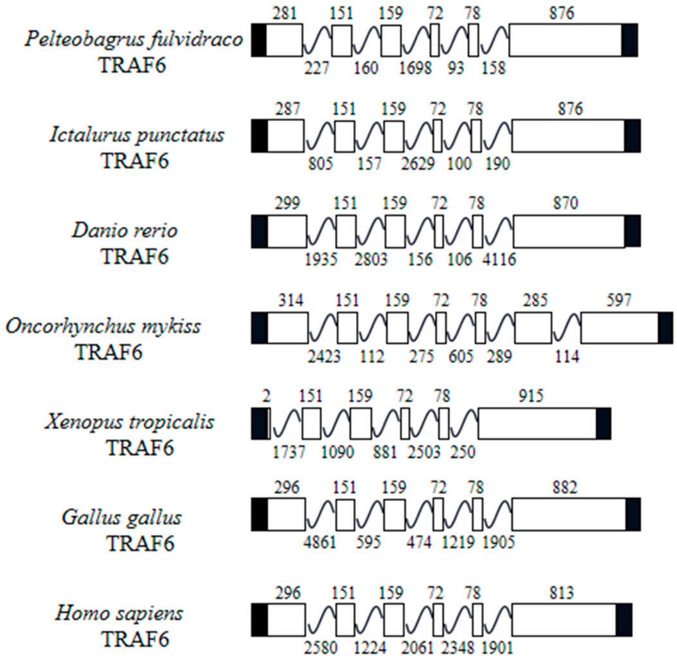

G:TRAF7

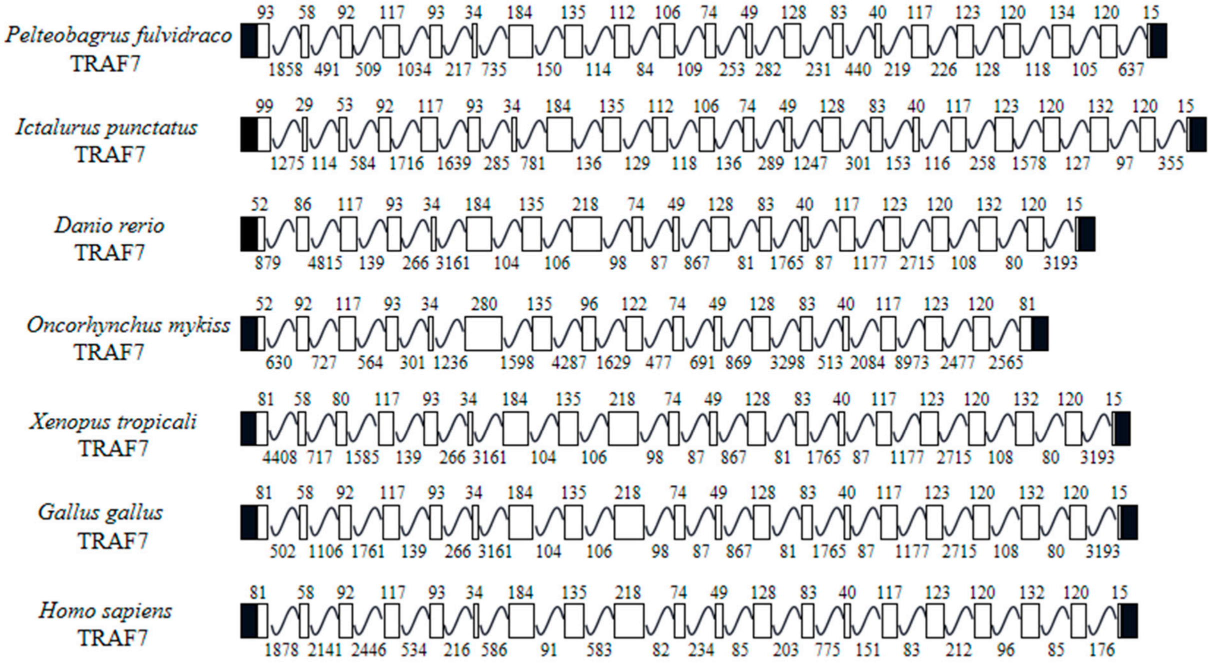
Pelteobagrus fulvidraco TRAF7
93 58 92 117 93 34 184 135 112 106 74 49 128 83 40 117 123 120 134 120 15
1858 491 509 1034 217 735 150 114 84 109 253 282 231 440 219 226 128 118 105 637
Ictalurus punctatus TRAF7
99 29 53 92 117 93 34 184 135 112 106 74 49 128 83 40 117 123 120 132 120 15
1275 114 584 1716 1639 285 781 136 129 118 136 289 1247 301 153 116 258 1578 127 97 355
Danio rerio TRAF7
52 86 117 93 34 184 135 218 74 49 128 83 40 117 123 120 132 120 15
879 4815 139 266 3161 104 106 98 87 867 81 1765 87 1177 2715 108 80 3193
Oncorhynchus mykiss TRAF7
52 92 117 93 34 280 135 96 122 74 49 128 83 40 117 123 120 81
630 727 564 301 1236 1598 4287 1629 477 691 869 3298 513 2084 8973 2477 2565
Xenopus tropicali TRAF7
81 58 80 117 93 34 184 135 218 74 49 128 83 40 117 123 120 132 120 15
4408 717 1585 139 266 3161 104 106 98 87 867 81 1765 87 1177 2715 108 80 3193
Gallus gallus TRAF7
81 58 92 117 93 34 184 135 218 74 49 128 83 40 117 123 120 132 120 15
502 1106 1761 139 266 3161 104 106 98 87 867 81 1765 87 1177 2715 108 80 3193
Homo sapiens TRAF7
81 58 92 117 93 34 184 135 218 74 49 128 83 40 117 123 120 132 120 15
1878 2141 2446 534 216 586 91 583 82 234 85 203 775 151 83 212 96 85 176
